# Development of TALE‐adenine base editors in plants

**DOI:** 10.1111/pbi.14246

**Published:** 2023-11-24

**Authors:** Dingbo Zhang, Jens Boch

**Affiliations:** ^1^ Institute of Plant Genetics Leibniz Universität Hannover Hannover Germany

**Keywords:** genome editing, plant breeding, rice, adenine deaminase, chloroplast

## Abstract

Base editors enable precise nucleotide changes at targeted genomic loci without requiring double‐stranded DNA breaks or repair templates. TALE‐adenine base editors (TALE‐ABEs) are genome editing tools, composed of a DNA‐binding domain from transcription activator‐like effectors (TALEs), an engineered adenosine deaminase (TadA8e), and a cytosine deaminase domain (DddA), that allow A•T‐to‐G•C editing in human mitochondrial DNA. However, the editing ability of TALE‐ABEs in plants apart from chloroplast DNA has not been described, so far, and the functional role how DddA enhances TadA8e is still unclear. We tested a series of TALE‐ABEs with different deaminase fusion architectures in *Nicotiana benthamiana* and rice. The results indicate that the double‐stranded DNA‐specific cytosine deaminase DddA can boost the activities of single‐stranded DNA‐specific deaminases (TadA8e or APOBEC3A) on double‐stranded DNA. We analysed A•T‐to‐G•C editing efficiencies in a β‐glucuronidase reporter system and showed precise adenine editing in genomic regions with high product purity in rice protoplasts. Furthermore, we have successfully regenerated rice plants with A•T‐to‐G•C mutations in the chloroplast genome using TALE‐ABE. Consequently, TALE‐adenine base editors provide alternatives for crop improvement and gene therapy by editing nuclear or organellar genomes.

## Introduction

The growing global population and the effects of climate change are challenging agricultural productivity. Genome editing by sequence‐specific nucleases such as meganucleases, zinc‐finger nucleases (ZFNs), transcription activator‐like effector nucleases (TALENs), and the CRISPR/Cas system has revolutionized genetic studies and crop breeding by enabling precise modifications of genomes (Gao, 2021). Such nucleases can induce double‐stranded DNA breaks (DSBs). In plants, the DSBs are predominantly repaired by non‐homologous end joining, which can generate random nucleotide insertions or deletions (indels) (Chen *et al*., 2019).

Many quantitative trait loci (QTLs) that drive crop production and stress tolerance are linked to single nucleotide polymorphisms (SNPs) (Huang and Han, 2014). Thus, the development of tools that can effectively cause single nucleotide variants instead of random indel mutations is essential. Base editors are genome editing technologies that can convert targeted base pairs without requiring DSBs or donor DNA templates (Anzalone *et al*., 2020). Two types of base editors have been developed: cytosine base editors (CBEs) convert C•G base pairs to T•A base pairs (Komor *et al*., 2016), and adenine base editors (ABEs) catalyse A•T‐to‐G•C conversions (Gaudelli *et al*., 2017). Typically, CBEs are composed of a CRISPR/Cas nickase, a cytosine deaminase, and a uracil glycosylase inhibitor (UGI). ABEs are comprised of a CRISPR/Cas nickase and an adenosine deaminase (Liu *et al*., 2022). An earlier version of ABE (ABE7.10) used a heterodimer of a wild‐type *E. coli* tRNA adenosine deaminase (TadA) and a synthetically evolved TadA (TadA7.10) to act on single‐stranded DNA (ssDNA) (Gaudelli *et al*., 2017). A further evolved TadA variant (TadA8e) exhibits improved editing efficiency and targeting scope in mammalian cells and rice (Richter *et al*., 2020; Wei *et al*., 2021; Yan *et al*., 2021). *In vitro* studies showed that TadA8e catalyses DNA deamination more than 1000‐fold faster than TadA7.10 (Lapinaite *et al*., 2020). When the Cas9 protein binds to its target DNA sequence, the sgRNA hybridizes to the complementary DNA sequence and causes an ssDNA R‐loop (Jiang and Doudna, 2017). This ssDNA exposure allows the ssDNA‐specific CBE and ABE deaminases to chemically modify their target bases within a window at the PAM‐distal end (Gu *et al*., 2021).

Recently, Mok et al. reported that the cytosine deaminase DddA_tox_ from *Burkholderia cenocepacia* enables targeted C•G‐to‐T•A conversions in human nuclear and mitochondrial double‐stranded DNA (dsDNA) (Mok *et al*., 2020). This enabled to use zinc finger or TALEs which do not cause ssDNA formation as targeting devices for the development of novel base editors (Lim *et al*., 2022; Mok *et al*., 2020, 2022a). TALEs can be placed more flexibly than Cas nucleases because they do not require the presence of a PAM sequence in a given distance to the target cytosine. DddA‐derived cytosine base editors (DdCBEs) use two split halves of DddA_tox_ (DddA‐N and DddA‐C) which are fused to two adjacent tail‐to‐tail TALE DNA‐binding arrays, respectively. The assembly of the two DddA halves reconstitutes the active enzyme which triggers deamination of target cytosines within the spacer region between the TALE binding sites (Mok *et al*., 2020). Such DdCBEs and ZF‐deaminases (ZFD) enabled base editing in organellar genomes, because they can be imported into organelles which is inefficient for CRISPR‐based systems (Lim *et al*., 2022; Mok *et al*., 2020). Accordingly, it is highly relevant to further expand and optimize such genome editing tools. In plants, DdCBEs were successfully used for editing the plastid genome of *Arabidopsis thaliana* (Nakazato *et al*., 2021), the chloroplast and mitochondrial DNA of lettuce, rapeseed (Kang *et al*., 2021), and rice chloroplasts (Li *et al*., 2021). Besides CBEs, TALE‐based ABEs (TALEDs) have recently also been developed to perform mitochondrial A•T‐to‐G•C base editing (Cho *et al*., 2022). These TALEDs are comprised of TALE DNA‐binding arrays, a full‐length DddA variant or split DddA, and an engineered deoxyadenosine deaminase (TadA8e) which catalyses the base conversion. When tested in 17 human mitochondrial target sites, TALEDs exhibited high editing efficiencies of up to 49% (Cho *et al*., 2022). Recently, it was reported that TALEDs could generate A•T‐to‐G•C base conversions in chloroplast DNA in lettuce protoplasts and *Arabidopsis* (Mok *et al*., 2022b). The DddA domain is essential for this editing system, but how DddA promotes TadA8e activity is still unknown. Neither TALE‐CBEs nor TALE‐ABEs have been applied for nuclear targets in plants, yet.

To easily compare different TALE‐base editor designs in plants, we developed a modular cloning (MoClo) pipeline for these tools and established a simple β‐glucuronidase (GUS) reporter assay in *Nicotiana benthamiana*. We present a series of TALE adenine base editors (TALE‐ABEs), compared their A•T‐to‐G•C editing activities, and demonstrate A•T‐to‐G•C editing in genomic loci in rice. Our experiments show that DddA enhances not only TadA8e but also other strictly ssDNA‐specific deaminases, which suggests that DddA somehow provides access to single‐stranded DNA. Moreover, to validate our TALE‐ABEs in the plant organelle genome, we targeted the rice chloroplast gene *OspsaA* and successfully generated chloroplast‐edited rice plants.

## Results

### Establishing a base editor reporter system with single TALE‐ABEs

**Figure 1:**
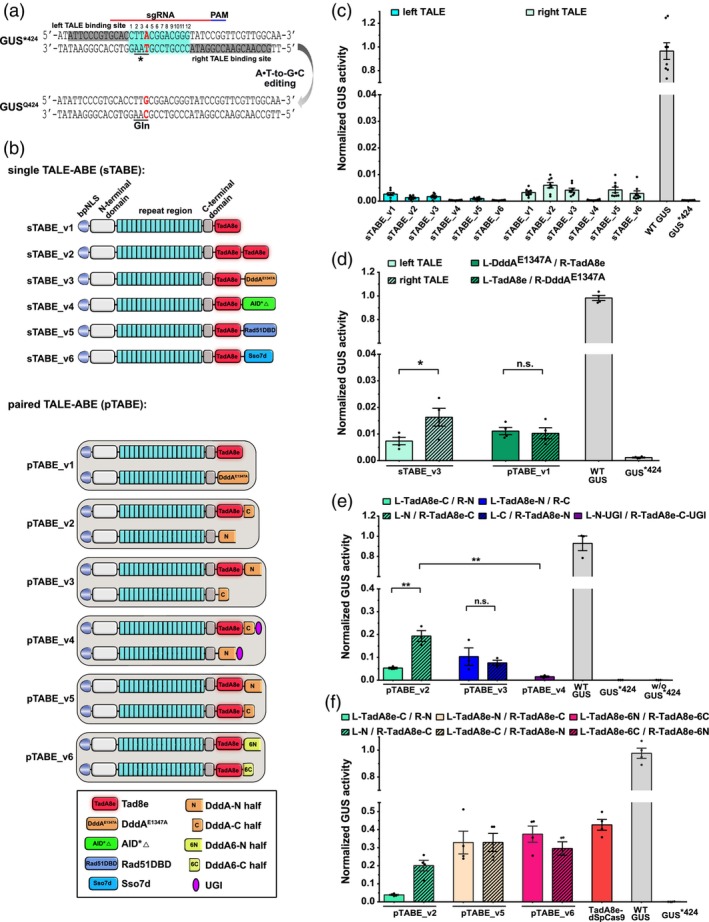
Establishment of TALE‐ABEs in *N. benthamiana*. (a) Schematic of the GUS*^424^ adenine base editing reporter. The A•T‐to‐G•C (highlight in red) editing in GUS*^424^ can alter the stop codon (TAA) to Gln (CAA) and restore GUS activity. TALE binding sites are in grey background, spacer region in cyan background, sgRNA targeting sequence and PAM are indicated by a red and blue line, respectively. (b) Architectures of six single TALE‐ABEs (sTABE_v1‐sTABE_v6) and six paired TALE‐ABEs (pTABE_v1‐pTABE_v6). bpNLS: bipartite nuclear localization signal; N/C: DddA‐N/DddA‐C halves split at G1397; 6N/6C: Ddd6A‐N/Ddd6A‐C halves split at G1397. (c–f) *A. tumefaciens* strains delivering constructs were mixed and infiltrated into *N. benthamiana* leaves. GUS activities were measured and normalized to 35S::GUS (WT GUS, positive control). Values are confirmed in independent experiments. (c) A•T‐to‐G•C editing efficiencies of six sTABEs binding to the left (left TALE) or right (right TALE) site in GUS*^424^, *n* = 8. (d) A•T‐to‐G•C editing efficiencies of sTABEs_v3 and pTABE_v1 binding to the left (L) or right (R) site in GUS*^424^, *n* = 4. (e) A•T‐to‐G•C editing efficiencies of pTABEs_v2, pTABE_v3, and pTABE_v4 at GUS*^424^ targeting sites, *n* = 3. (f) A•T‐to‐G•C editing efficiencies of three pTABEs (pTABEs_v2, pTABE_v5, and pTABE_v6) and TadA8e‐dSpCas9 at GUS*^424^ targeting sites, *n* = 4. GUS*^424^: negative control, w/o GUS (without GUS*^424^, pTABEs only): negative control. Values and error bars indicate the mean ± SEM, *P < 0.05; **P < 0.01; n.s. (not significant) using Student's two‐tailed unpaired *t*‐test.

We first developed a β‐glucuronidase (GUS) reporter system in *N. benthamiana* for evaluating A•T‐to‐G•C editing efficiencies of different base editors. For this, an inactivated GUS variant (GUS*^424^) was constructed containing a stop‐codon (TAA). An A•T‐to‐G•C conversion in the non‐coding strand can revert the stop codon to a codon encoding glutamine (CAA) and facilitates the production of functional GUS protein (Figure 1a). The GUS activity can then be used as approximation for the efficiency of base editing.

Next, we employed two separate strategies for the construction of TALE‐ABEs (Figure 1b). One is using a single TALE array containing a bipartite nuclear localization sequence (bpNLS), an N‐terminal TALE domain, the RVD repeat region, the C‐terminal TALE domain, and functional domains to perform the base editing, termed single TALE‐ABE (sTABE). The other is using a pair of TALE‐ABEs and we named them paired TALE‐ABE (pTABE). To enable a simple construction of different base editor designs, individual parts were built as modular cloning (MoClo) modules and assembled using Golden Gate Cloning (Figure S1). First, TadA8e or a TadA8e‐dimer was fused to the TALE array to generate sTABE_v1 and sTABE_v2. The activity of these sTABEs was tested using the GUS*^424^ reporter by *Agrobacterium*‐mediated transient expression in *N. benthamiana* leaves (Figure S2). After normalization to a constitutively expressed functional GUS (WT GUS), both sTABEs conferred very low GUS activity (0.2%–0.6%) with the sTABE‐binding either left (non‐coding strand, shown as L) or right (coding strand, shown as R) of the target adenine in the GUS*^424^ reporter (Figure 1a,c).

Next, we examined whether fusing additional domains to the sTABE can increase their A•T‐to‐G•C editing efficiency. Cho *et al*. reported that the dsDNA‐specific cytosine deaminase domain of DddA as catalytic inactive (DddA^E1347A^) or active version in different designs drastically enhances the activity of TALE adenine base editors on human mitochondrial DNA (Cho *et al*., 2022). Accordingly, DddA^E1347A^ was fused to the single TadA8e and yielded sTABE_v3 (Figure 1b). sTABE_v4 harbours an engineered human AID (AID*Δ) which exhibited high deaminase activity in rice (Ren *et al*., 2018). sTABE_v5 contains the single‐strand DNA‐binding domain from RAD51 protein (Rad51DBD) which conferred increased activity in cytosine base editors (Zhang *et al*., 2020) and adenine base editors (Tan *et al*., 2022). sTABE_v6 contains the non‐specific double‐strand DNA‐binding protein Sso7d from *Sulfolobus solfataricus* (Baumann *et al*., 1995). Overall, all setups with the exception of sTABE_v4 showed comparable GUS activity above background with slight preferences for binding either left or right of the target site (Figure 1c). These results show that TadA8e can catalyse A•T‐to‐G•C editing in dsDNA in sTABE architectures, but the editing efficiencies are very low. Fusion of DddA^E1347A^, AID*Δ, Rad51DBD, or Sso7d could not increase the editing efficiency in our reporter assays.

### Improving the A•T‐to‐G•C editing efficiency with paired TALE‐ABEs

As an alternative design, we tested paired TALE‐ABE (pTABE) architectures to combine TadA8e and DddA. The pTABEs (pTABE_v1 to pTABE_v6) are composed of a pair of TALE arrays in a tail‐to‐tail arrangement (Figure 1b). For pTABE_v1, TadA8e was fused to one TALE array and the catalytically inactive DddA^E1347A^ was fused to the other. The editing efficiencies of pTABE_v1 was comparable to the single TALE‐ABE (sTABE_v3) (Figure 1d). The activity of the single sTABE_v3 varied between 0.7% and 1.6% when positioned on the left or right side of the target adenine, respectively. The activity of the pair pTABE_v1 was similar (1%) for both orientations, but remained low (Figure 1d). This indicates that the fusion of a full‐length DddA^E1347A^ in sTABE or pTABE could not increase the TALE‐ABE A•T‐to‐G•C editing efficiency in our *N. benthamiana* assay.

Next, we tested split DddA (split at G1397) designs with the DddA‐N and DddA‐C halves fused separately to a pair of TALE arrays with or without the addition of an uracil‐glycosylase inhibitor (UGI) (pTABE_v2, pTABE_v3, pTABE_v4). Remarkably, two of these designs showed significantly increased base editing activity. When DddA‐C and TadA8e were fused to one TALE array and DddA‐N to the other (pTABE_v2), the activities varied between 5.3% and 19.3% depending on the orientation of the pTABE pair (Figure 1e). If the position of the two DddA‐halves in the fusion constructs was switched (pTABE_v3), the activities ranged from 7.6% to 10.3% (Figure 1e). In contrast, the activity of pTABE_v4 which has the same architecture as pTABE_v2 but harbours additional UGIs following the DddA‐C and DddA‐N halves was significantly lower (1.5%).

We speculated that the spatial position of TadA8e to the target adenine might influence editing efficiency and that a single adenine deaminase might not be optimal. To alleviate this, we added TadA8e to both TALE arrays in addition to one of the DddA halves (pTABE_v5). Indeed, pTABE_v5 exhibited a significantly higher activity (32.9%) than pTABE_v2, irrespective of the orientation of the pair (Figure 1f). Finally, we used the DddA variant DddA6 which has been reported to exhibit an increased catalytic activity (Mok *et al*., 2022a) as DddA6‐N and DddA6‐C halves (pTABE_v6). pTABE_v6 showed a slightly higher activity (37.5%) in one orientation, but a slightly lower one (29.6%) in the other orientation which was comparable to the normal DddA halves.

To compare the efficiencies of the TALE‐base editors with CRISPR/Cas9 editors, we designed an sgRNA for the target sequence (Figure 1a) and used a catalytically dead dSpCas9‐adenine base editor (TadA8e‐dSpCas9) targeting the GUS*^424^ reporter. We chose dCas9 instead of a Cas9‐nickase for a fair comparison, because TALE‐base editors are also not able to guide repair via nicking of the non‐edited DNA strand. The activity of this CRISPR/Cas9 base editor was slightly higher (42.7%), but in a similar range than the two TALE‐base editors (pTABE_v5, pTABE_v6) (Figure 1f).

These results indicate that split‐DddA but not full‐length DddA^E1347A^ could increase A•T‐to‐G•C editing in TALE‐ABEs in our *N. benthamiana* reporter system. Moreover, paired TALE arrays containing TadA8e on both TALEs and split‐DddA or DddA6 halves can further increase editing efficiency.

### pTABE_v4 allows C•G‐to‐T•A editing

**Figure 2:**
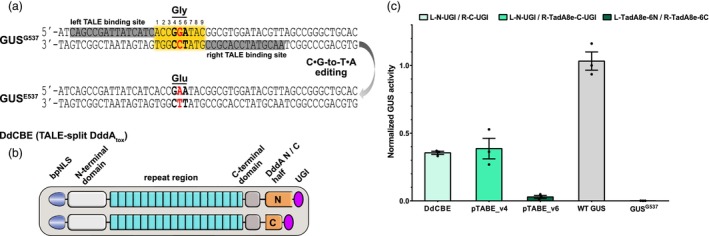
Efficient C•G‐to‐T•A editing occurs only in the presence of UGI. (a) Schematic of the GUS^G537^ cytosine base editing reporter. The inactive GUS^G537^ contains Gly (GGA) at position 537. C•G‐to‐T•A (highlight in red) editing of GUS^G537^ can change the Gly to Glu (GAA) and restore GUS activity. Left and right TALE binding sites in grey background, spacer region in orange background. (b) Architectures of TALE‐split DddA_tox_ (DdCBE) to target GUS^G537^. (c) C•G‐to‐T•A editing efficiencies of the cytosine base editor DdCBE, paired adenine base editors with (pTABE_v4) and without (pTABE_v6) UGI. GUS activities were determined from *A. tumefaciens‐*infiltrated *N. benthamiana* leaves and normalized to 35S::GUS (WT GUS). Values and error bars indicate the mean ± SEM, *n* = 3.

Because some pTABEs contain split DddA halves which are in principle catalytically active, we wondered whether these pTABEs can also edit cytosines. Hence, we developed a GUS reporter (GUS^G537^) with a missense mutation that converts an amino acid in the active centre of the enzyme from glutamate to glycine to abrogate its activity (Islam *et al*., 1999). Cytosine base editing can revert this mutation to restore the GUS activity. The target site contains a TC motif which is required for DddA activity (Figure 2a).

We then constructed TALE‐CBEs (DdCBEs) that resembled the original design of Mok *et al*. with split DddA_tox_ halves and UGI fused to each TALE array (Mok *et al*., 2020) as a positive control (Figure 2b). By utilizing the same left and right TALE arrays, the DdCBEs and pTABE_v4 (containing UGI) showed similar GUS activity of average 35.5% and 38.6%, whereas pTABE_v6 (without UGI) showed a very low GUS activity of 2.9% (Figure 2c). This indicates that the addition of UGI to paired TALE‐ABEs (pTABE_v4) enables efficient C•G‐to‐T•A conversion; however, in the absence of UGI (pTABE_v6), this conversion is very inefficient.

### DddA^E1347A^ makes dsDNA accessible for ssDNA‐specific deaminases

**Figure 3:**
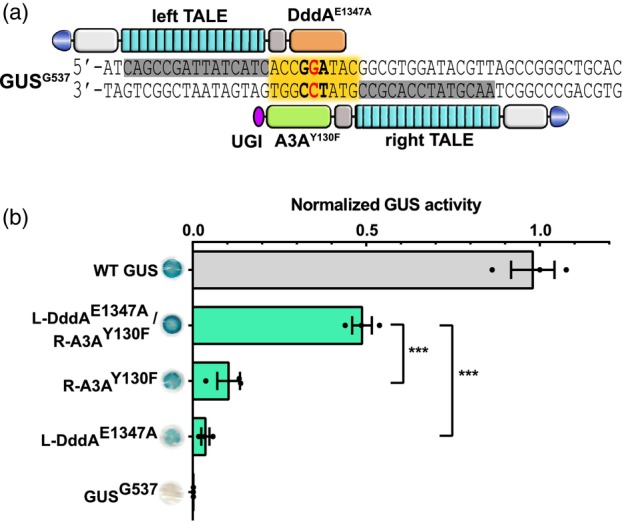
DddA enables efficient base editing of APOBEC3A. (a) Schematic of the DddA^E1347A^/A3A^Y130F^ cytosine base editing system targeting the GUS^G537^ cytosine base editing reporter. Left TALE fused with DddA^E1347A^, right TALE fused with APOBEC3A (A3A^Y130F^). (b) C•G‐to‐T•A editing efficiencies of cytosine base editors. One representative stained leaf disk of the qualitative assay is shown. GUS activities were determined from *A. tumefaciens‐*infiltrated *N. benthamiana* leaves and normalized to 35S::GUS (WT GUS). Values and error bars indicate the mean ± SEM, *n* = 3. ***P < 0.001 using Student's two‐tailed unpaired *t*‐test.

It was puzzling why the ssDNA‐specific TadA8e could efficiently use a dsDNA substrate when fused to DddA‐N and DddA‐C halves. Our hypothesis was that the DddA acts on dsDNA, e.g., by partially unwinding the double strand and revealing ssDNA locally. To address this, we tested the activity of another highly ssDNA‐specific deaminase, the cytosine deaminase human APOBEC3A (A3A), using our cytosine GUS^G537^ reporter (Figure 3a). For this, DddA^E1347A^ was fused to the left TALE array and the highly active A3A variant A3A^Y130F^ (Ren *et al*., 2021; Zhou *et al*., 2019) was fused to the right TALE array. A3A only exhibits cytosine deaminase activity against ssDNA (Moraes *et al*., 2021) while DddA^E1347A^ has no cytosine deaminase activity against both, dsDNA and ssDNA (Mok *et al*., 2020). When targeting the GUS^G537^ reporter with DddA^E1347A^/A3A^Y130F^ CBEs in *N. benthamiana* GUS assays, the combination of DddA^E1347A^ and A3A^Y130F^ CBEs exhibited a very high GUS activity of 48.7%, while A3A^Y130F^ and DddA^E1347A^ alone only show 10.3% and 3.6% GUS activity, respectively (Figure 3b). This suggests that DddA^E1347A^ generally makes target bases in dsDNA more accessible for local ssDNA‐specific deaminases, possibly by partially unwinding the dsDNA.

### The spatial requirement of paired TALE‐adenine base editors

**Figure 4:**
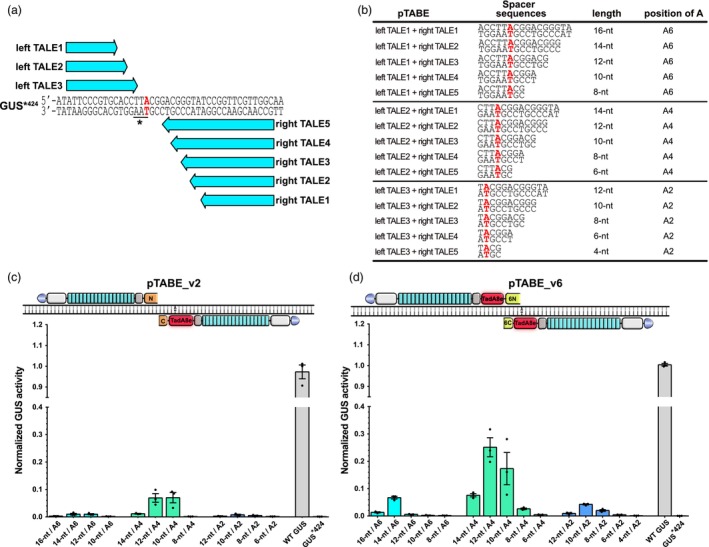
Analysing the editing windows of paired TALE‐ABEs. (a) Schematic of shifting the editing window of paired TALE‐ABEs (pTABEs) and the position of the target adenine by using TALE arrays of different length; the binding sites of three left TALEs and five right TALEs are show by blue arrows. The targeted A•T base pair is in red. (b) Different spacer regions (from 4 to 16) flanked by different left and right TALE combinations. The targeted adenine is in red and bold. (c, d) A•T‐to‐G•C editing efficiencies of pTABE_v2 (c) and pTABE_v6 (d) in the GUS*^424^ reporter. Top: architecture of pTABE_v2 or pTABE_v6, left TALE binding to the non‐coding strand of GUS*^424^ and right TALE binding to the coding strand. Bottom: values and error bars indicating the mean ± SEM, *n* = 3. GUS activities were determined from *A. tumefaciens*‐infiltrated *N. benthamiana* leaves and normalized to 35S::GUS (WT GUS).

To apply base editors, it is crucial to know the editing window, i.e., the target region where the deaminase is acting, relative to the DNA‐binding site of the tool. Previously, this has been studied for TALE‐base editors by amplicon sequencing of target regions which revealed the editing efficiencies of different possible bases in the regions (Mok *et al*., 2020, 2022a). In contrast, we aimed to understand how a pair of TALE‐ABEs should be positioned to modify a specific target base. Therefore, we constructed TALE arrays of different lengths flanking the target adenine in the GUS*^424^ reporter (Figure 4a). The different combinations of left and right TALEs allow to test different sizes of spacer regions (from 4 to 16 nucleotides; 4‐nt to 16‐nt), and to vary the position of the targeted adenine in the spacer (from position 2–6; A2–A6) (Figure 4b). We tested the editing efficiencies of pTABE_v2 (with DddA‐halves; Figure 4c) and pTABE_v6 (with DddA6‐halves; Figure 4d) with different TALE combinations separately in *N. benthamiana* GUS assays. Across the 6‐nt to 16‐nt spacers, pTABE_v2 achieved the highest editing efficiency at position A4 in the 10‐nt and 12‐nt spacer regions, and the A4 editing activities are significantly reduced when the spacer is extended to 14‐nt or shortened to 8‐nt. When the targeted adenine was located at A2 or A6, pTABE_v2 showed poor editing activities. Similarly, pTABE_v6 also showed high activity at A4 in the 10‐nt to 12‐nt spacer regions. In addition, pTABE_v6 still showed significant activities at A6 in the 14‐nt spacer and A2 in the 10‐nt spacer. These results indicate that both pTABE_v2 and pTABE_v6 prefer target adenines located at A4 in a spacer region of 10–12 nucleotides.

### Refining the editing range in the spacer region of paired TALE‐base editors in plant protoplasts

**Figure 5:**
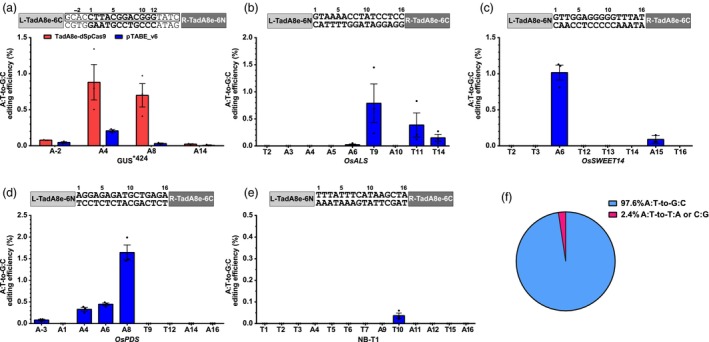
Editing efficiency of pTABE_v6 in rice and *N. benthamiana*. (a–e) A•T‐to‐G•C editing efficiencies were determined by amplicon sequencing of target regions from the *A. tumefaciens‐*infiltrated GUS*^424^ ABE reporter (a) or transformed rice (b–d) and *N. benthamiana* (e) protoplasts. Targeted sequences are listed above the panels. Spacer sequences are in bold. sgRNA for TadA8e‐dSpCas9 is indicated by a rectangle. Blue: pTABE_v6, red: TadA8e‐dSpCas9. (f) Product purities of pTABE_v6 from (b–e). Values and error bars indicate the mean ± SEM, *n* = 3.

More than one adenine might be edited in the spacer region of paired TALE‐base editors, in particular, if both DNA strands could potentially be targeted. To reveal the editing range of pTABE_v6 in comparison to TadA8e‐dSpCas9, we amplified the target region in the GUS*^424^ reporter from DNA of *N. benthamiana* leaves infiltrated with *Agrobacterium* strains co‐delivering the GUS reporter and base editor components and sequenced the amplicons by next‐generation sequencing. Both pTABE_v6 and TadA8e‐dSpCas9 showed the highest adenine editing activity at position A4 (which restored the stop‐codon to glutamine) with average 0.2% and 0.9% editing efficiencies, respectively (Figure 5a). In addition, TadA8e‐dSpCas9 showed 0.1% and 0.7% efficiencies at A2 and A8, respectively, while pTABE_v6 showed very low editing (<0.1%) at these sites.

To further characterize the targeting capabilities of TALE‐ABEs on plant nuclear loci, we used pTABE_v6 to target three chromosomal rice genes (*OsALS*, *OsSWEET14*, and *OsPDS*) and one chromosomal *N. benthamiana* gene (NB‐T1) in protoplasts. Amplicon sequencing showed that in the 16‐nt spacer region of *OsALS* three A•Ts were edited (T9, T11, and T14) with efficiencies from 0.2% to 0.8% (Figure 5b). In *OsSWEET14*, two A•Ts were edited with an efficiency of 1% for A6 and 0.1% for A15 (Figure 5c). In *OsPDS*, four A•Ts were edited with the highest editing efficiency of 1.5% at A8 (Figure 5d). In NB‐T1, only very low editing was detectable at T10 (Figure 5e). The average editing product purity of pTABE_v6 reached 97.6% with 2.4% transversions to C or T, and we did not identify any C•G‐to‐T•A editing or indels in those five target sites (Figure 5f, Data S1). These results show that pTABE_v6 generates A•T‐to‐G•C conversions with high product purity and can target chromosomal loci in plant cells.

### Off‐target editing by paired TALE‐ABEs

TALE‐CBEs directed to the mitochondria have been reported to result in off‐target editing in mitochondria and even the nuclear chromosomes (Lee *et al*., 2022; Lei *et al*., 2022; Mok *et al*., 2022a). This off‐target editing appears puzzling, given that DNA recognition by TALE arrays is considerably specific and the requirement for two neighbouring binding sites makes TALEN pairs explicitly specific. One possibility is that the interaction between the two DddA halves is strong enough to enable reconstitution of the functional deaminase even if only one TALE array is bound to an off‐target site.

**Figure 6:**
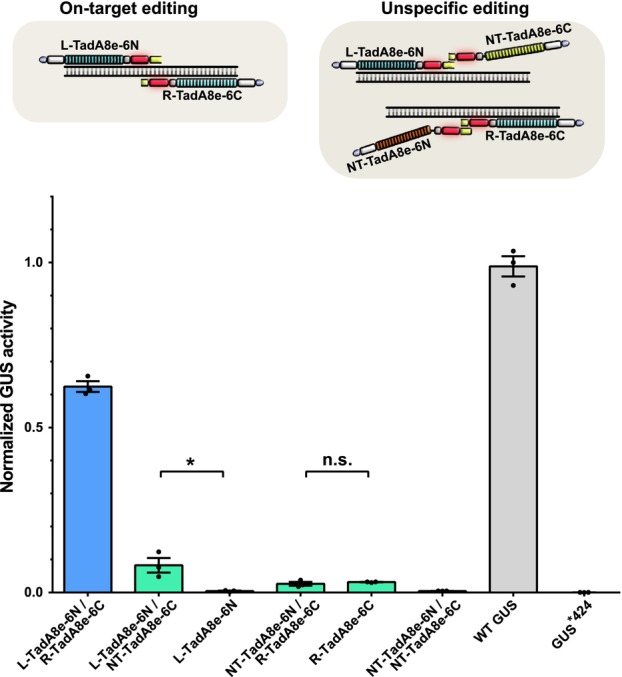
Off‐target editing of pTABE_v6. A•T‐to‐G•C editing efficiencies by pTABE_v6 with a pair of targeted pTABEs (L‐TadA8e‐6N/R‐TadA8e‐6C) or a combination of targeted and non‐targeted TABE (L‐TadA8e‐6N/NT‐TadA8e‐6C or NT‐TadA8e‐6N/R‐TadA8e‐6C). GUS activities were determined from *A. tumefaciens‐*infiltrated *N. benthamiana* leaves and normalized to 35S::GUS (WT GUS). Bottom: values and error bars indicate the mean ± SEM, *n* = 3. *P < 0.05; n.s. (not significant) using Student's two‐tailed unpaired *t*‐test.

To profile the off‐target editing of our paired TALE‐ABEs, we designed two pairs of TALE arrays based on pTABE_v6 with one TALE array binding to the target site in the GUS*^424^ reporter (shown as L‐TadA8e‐6N or R‐TadA8e‐6C, Figure 6) and the other one containing non‐targeted RVD repeats that cannot bind to the target sites (shown as NT‐TadA8e‐6N or NT‐TadA8e‐6C, Figure 6). Determining base editing activity in *N. benthamiana* GUS assays, we found that the combination of L‐TadA8e‐6N/NT‐TadA8e‐6C showed an average of 8.3% GUS activity (positive control is 62.4%, L‐TadA8e‐6N/R‐TadA8e‐6C) while L‐TadA8e‐6N alone (without the NT‐TadA8e‐6C) showed a background activity of only 0.4%. This indicated that there is a considerable editing if only one of the two paired TALE‐ABEs is bound to the target site. If the DddA‐halves are switched, the combination of NT‐TadA8e‐6N/R‐TadA8e‐6C or the single R‐TadA8e‐6C alone both lead to about 3% editing activity (positive control is 62.4%). This shows that one of the paired TALE‐ABEs can trigger low‐level off‐target editing when bound to a DNA location.

### pTABE_v6 mediated A•T‐to‐G•C conversions in rice chloroplast

**Figure 7:**
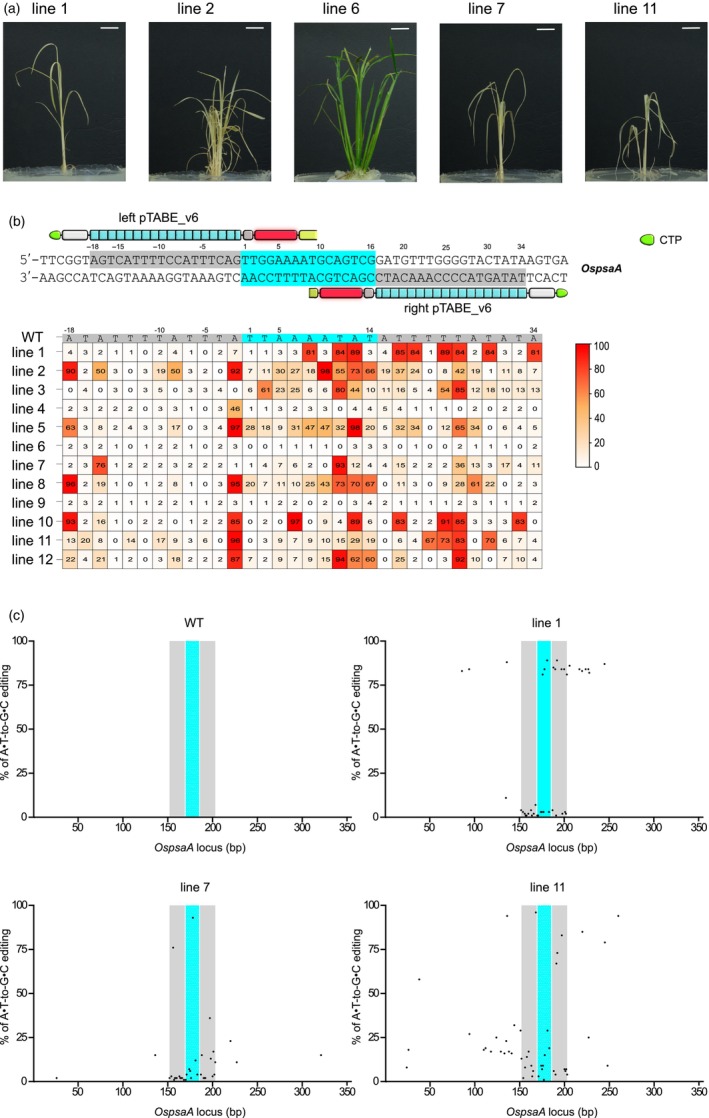
pTABE_v6‐induced chloroplast genome editing in rice. (a) Phenotypes of five representative transgenic lines grown in rooting medium. Bar = 1 cm. (b) Top: paired pTABE_v6 targeting the *OspsaA* chloroplast gene in rice. Bottom: heat map showing A•T‐to‐G•C conversions at the *OspsaA* site from 12 regenerated rice plants. CTP, chloroplast transition peptide. (c) Investigation of the off‐target effects caused by pTABE_v6 in three representative transgenic lines. Dots showing the A•T‐to‐G•C editing frequencies in relation to the reference sequence and the position ±150 bp spanning the *OspsaA* target site. Left and right TALE binding sites are indicated in grey background, the spacer region is indicated in cyan background. WT, wild‐type. The editing efficiencies in (b and c) are analysed by EditR for quantification.

To demonstrate the efficiency of the TALE‐ABE systems in converting A•T‐to‐G•C in rice organelles, we targeted the chloroplast gene *OspsaA*. Mutations in the *OspsaA* gene result in an albino phenotype due to the decreased chlorophyll production (Li *et al*., 2021). We found that 10 out of 12 regenerated lines exhibited the albino phenotype (Figure 7a and Figure S3). Sanger sequencing results showed A•T‐to‐G•C mutations within the spacer region of the albino lines, but not in the green lines (Figure S4). To further quantify the A•T‐to‐G•C editing efficiencies, we used the EditR webtool (Kluesner *et al*., 2018) to evaluate the rates of base editing from the sequencing chromatograms (Figure 7b). As a result, we found multiple A•T‐to‐G•C conversions with editing frequencies of up to 98% within the spacer region in the 10 albino lines. While the two non‐albino lines, line 6 and line 9, showed nearly no A•T‐to‐G•C conversion. We noticed that the pTABE_v6 also induced high A•T‐to‐G•C conversions at the TALE binding sites in the albino lines. This bystander editing, caused by TALE‐ABE, has also been reported in mammalian cells (Cho *et al*., 2022). To investigate the unexpected A•T‐to‐G•C editing, we further analysed the 150 bp regions flanking the TALE binding sites in the albino lines (Figure 7c and Figure S5). Unexpectedly, several A•T‐to‐G•C mutations with various conversion frequencies were found in those regions, suggesting that pTABE_v6 can induce off‐target editing in the chloroplast genome. We did not identify any C•G‐to‐T•A editing in those lines, which shows that DddA cannot induce C•G‐to‐T•A changes without UGI. Taken together, these results demonstrate that pTABE_v6 can generate nearly homoplasmic A•T‐to‐G•C editing in rice chloroplasts, albeit the presence of off‐target editing.

## Discussion

In the present study, we tested two different designs of TALE‐adenine base editors, single TALE‐ABE editors and paired TALE‐ABE editors, and refined how to apply them in plant cells on nuclear target sequences. This work extends the initial description of efficient A•T‐to‐G•C editing via TALE‐based genome editing tools (Cho *et al*., 2022). Compared to the previous studies in human mitochondrial DNA (Cho *et al*., 2022), we tested more combination of TALE‐ABE architectures and found two superior variants. In plant cells, only one version of TALE‐ABE architectures has been tested, so far (Mok *et al*., 2022b). In this study, we established GUS reporter and scored adenine base editing in the plant nucleus and chloroplasts. To accomplish a quick assembly of different tool designs, we based all components on a modular MoClo design.

Our results confirm that fusion of DddA to the adenine deaminase is crucial to achieve a high adenine editing efficiency, also in plant cells. For this, we found that fusion of split halves of DddA or the enhanced DddA6 variant (Mok *et al*., 2022a) to the TadA8e adenine deaminase variant (Richter *et al*., 2020) is most effective compared to other protein fusion strategies. A spacer length of 10–12 nt is optimal, with the targeted adenine at position 4. Previous studies have shown that TadA8e has a higher DNA deaminating ability than other TadA variants. Although TadA8e cannot access dsDNA, it is *in vitro* capable of rapidly deaminating transiently generated single‐strand DNA that might occur during the search process of CRISPR/Cas‐systems for target sites (Lapinaite *et al*., 2020). Accordingly, TadA8e fused to a TALE array alone only resulted in very low adenine editing in our assay which confirms that TadA8e itself cannot act efficiently on dsDNA. In contrast, our experiments now support a model that the dsDNA‐specific DddA provides access to ssDNA. A TALE‐DddA^E1347A^ fusion enabled efficient base editing by the ssDNA‐specific cytosine deaminase APOBEC3A (A3A^Y130F^) when fused to a TALE array. A possible explanation is that DddA unwinds dsDNA locally to facilitate its own cytosine deaminase activity. Similarly, the catalytically dead DddA^E1347A^ variant transiently provides ssDNA as a substrate for A3A^Y130F^ and the split DddA variants enabled TadA8e activity in our reporter assays. We noticed that the overall activity of the paired CBE is very high in comparison to the low activity of paired ABEs using full‐length DddA^E1347A^ (compare Figure 3b and pTABE_v1 in Figure 1d). This inconsistency might be caused by the differences in the CBE‐reporter and ABE‐reporter as well as the catalytic domains involved. Nevertheless, it indicates that DddA can support the activity of ssDNA‐specific enzymes in different designs. Furthermore, DddA and DddA6 are limited to a 5’‐TC context for cytosine base editing (Mok *et al*., 2020, 2022a); however, split DddA and DddA6 in our pTABEs do not require a 5′‐TC motif to facilitate adenine base editing of TadA8e, which is consistent with the previous study in mammalian cell lines (Cho *et al*., 2022). Taken together, this opens up interesting questions regarding the mechanistic details whether and how dsDNA‐specific cytosine deaminases like DddA possibly target DNA bases in a two‐step process of unwinding DNA and subsequent base deamination.

Cho *et al*. showed an efficient A•T‐to‐G•C conversion in human mitochondrial DNA via monomeric TALEDs (similar architecture as sTABE_v3), dimeric TALEDs (similar architecture as pTABE_v1), and split TALEDs (similar architecture as pTABE_v2) (Cho *et al*., 2022). In contrast, in our GUS reporter assays, both sTABE_v3 and pTABE_v1 show only low activity compared to pTABE_v2. It is worth noticing that there is a difference how the base modification is fixed in human mitochondria and in the *N. benthamiana* transient expression system, respectively. The circular mitochondrial DNA in a multiplying cell culture is replicating quickly, which fixes heteroduplex situations into mutations in one of the daughter molecules without the need for a DNA repair process. This enhances mutational changes by TALE‐base editors that do not nick DNA and which in contrast to CRISPR/Cas9 base editors (Komor *et al*., 2016) are unable to guide the replacement of the non‐edited DNA strand. Furthermore, amplicon sequencing can overestimate base editing mutation rates, because also transient, non‐resolved heteroduplexes are amplified and scored as mutations. In our GUS assay, the base deaminases target a base in the template strand which is directly transcribed into the desired modification even if a heteroduplex still exists, which also potentially enhances the apparent editing. The target adenine in our GUS reporter is part of a TA motif which is favoured by TadA8e (Wu *et al*., 2022). At the same time, we can only detect TAA to CAA edits in the GUS*^424^ reporter and other base edits are not detected which might result in an underestimation of total editing rates.

So far, TALE‐base editors have achieved near‐homoplasmic editing rates in chloroplasts of full organisms, namely *Arabidopsis*, lettuce, and rice (Kang *et al*., 2021; Li *et al*., 2021; Mok *et al*., 2022b; Nakazato *et al*., 2021). In this study, we were also able to achieve nearly homoplasmic A•T‐to‐G•C editing in chloroplasts of regenerated rice plants. However, at present no targeted editing in the nuclear chromosomes by TALE‐base editors has been reported. Although we achieved high editing rates in our reporter system which in principle is nuclear localized, and we detected editing of rice and *N. benthamiana* chromosomal loci, we were not able to regenerate any T_0_ plants with edited nuclear loci although we targeted the same sites that were successfully edited in protoplasts. Either the base changes are not fixed efficiently in the nuclear chromosome without nicking of the non‐edited strand or TALE‐base editors have an overall deleterious effect on the cell, e.g., by high off‐target rates, which hinders plant regeneration. Recent studies presented a novel TALE base editor named mitoBEs, which fused a nickase (MutH or Nt.BspD6I(C)) and a deaminase (adenine deaminase TadA8e or cytosine deaminase APOBEC1) to a pair of TALEs, respectively (Yi *et al*., 2023). MitoBEs exhibited A•T‐to‐G•C or C•G‐to‐T•A editing with an efficiency of up to 77% and high specificity in human cells while targeting mitochondrial DNA (Yi *et al*., 2023). It will be interesting to test the nickase in our TALE‐ABEs, as this may lead to improved specificity in A•T‐to‐G•C editing also in the organellar genome of plants.

Substantial nuclear off‐target editing of TALE‐CBEs has been reported even when the tool was directed to the mitochondria (Lei *et al*., 2022). The two DddA halves were speculated to associate to a functional enzyme even at sites where only one TALE array is bound. We could confirm that the activity of our pTABE_v6 editor can be detected when only one TALE array is binding the target locus. Apparently, spontaneous re‐association of the DddA halves promotes this. The use of an engineered low‐off target DddA (Lee *et al*., 2022; Lei *et al*., 2022) might alleviate this. Also, in particular the TadA8e variant causes elevated off‐target editing in plant genomes when used in CRISPR/Cas9 base editors (Wu *et al*., 2022). Possibly, this editing is linked to transient ssDNA areas caused by the Cas9 target search and would not appear by TALE‐TadA8e tools.

In this study, we found that pTABE_v6 can generate substantial A•T‐to‐G•C off‐target editing along the TALE binding regions in rice chloroplasts. However, this off‐targeting effect was not detected in our rice protoplast assays or the *N. benthamiana* transient expression system. We hypothesize that chloroplasts are specifically prone to off‐target editing, because ssDNA is transiently formed during replication of the multiple genome copies within a chloroplast. This ssDNA can efficiently be deaminated by TadA8e which has a rapid deaminase kinetic (Lapinaite *et al*., 2020). In addition, pTABE_v6 is constitutively expressed during regeneration of the plants causing a much prolonged editing window in comparison to protoplast assays or GUS reporter studies. To reduce the unspecific editing by TadA8e, highly specific TadA8e variants (Chen *et al*., 2023; Jeong *et al*., 2021) can be utilized in TALE‐ABEs.

In summary, we have refined the optimal architecture of the TALE‐adenine base editing system in plant cells. This system can now be applied for mitochondrial and chloroplast genome editing to accelerate crop improvement. Future work will address the efficiency for nuclear chromosomal editing.

## Methods

### Plasmid construction

We used the modular cloning (MoClo) syntax (Geiβler *et al*., 2011; Grützner and Marillonnet, 2020; Weber *et al*., 2011) to generate the TALE‐ABE plasmids. For this, the components were subcloned in individual modules that can be assembled using Golden Gate Cloning (Engler *et al*., 2008). The details of the cloning procedures are listed in Method S1 and Figure S1. The plasmid modules used in this study were listed in Table S1.

### Plant growth condition

*Nicotiana benthamiana* plants were grown in a greenhouse with 16 h of light, a relative humidity of 40%–60%, and temperatures of 23 °C and 19 °C during the day and night, respectively. Four‐ to six‐week‐old plants were used for *Agrobacterium tumefaciens* inoculation experiments.

### *Nicotiana benthamiana* infiltration and GUS reporter assay

GUS reporter assays were performed as previously described (Boch *et al*., 2009). Briefly, *A. tumefaciens* GV3101 strains containing a TALE‐ABE construct, the GUS reporter construct, and a p19 silencing inhibitor, respectively, were mixed 1:1:1 with OD_600_ of 0.8 and inoculated into *N. benthamiana* leaves. Two to three days after inoculation, two leaf discs (diameter 0.8 cm) were harvested from each inoculation spot. Leaf tissues were homogenized and incubated with 4‐methyl‐umbelliferyl‐β‐D‐glucuronide. GUS activities were measured using a TECAN reader (360 nm excitation and 465 nm emission). For details see Figure S2. Proteins were quantified by NanoDrop™ One (Thermo Fisher Scientific). Leaf disks were stained in X‐Gluc solution and de‐stained in ethanol.

### Protoplast isolation and transformation

We used rice cultivar Kitaake leaves to prepare rice protoplasts. Protoplast isolation and transformation were performed as previously described (Shan *et al*., 2014). 10 μg plasmid DNA per construct were introduced into protoplasts by PEG‐mediated transfection. The transfected protoplasts were incubated at room temperature. After 48 h, the protoplasts were collected and the genomic DNA extracted.

### Rice stable transformation with TALE‐ABE constructs

The *japonica* rice cultivar Kitaake was used for *A. tumefaciens*‐mediated stable transformation as previously described (Sallaud *et al*., 2003). Briefly, two *A. tumefaciens* strains EHA105, each containing one of the two pTABE_v6, were mixed (1:1) prior to transformation of calli. Then the calli were transferred to plates containing 50 mg/l hygromycin for selection. Regenerated rice plants were subjected to phenotyping and genotyping (Figure S3a). The Sanger sequencing results of pTABE_v6 were analysed and quantified using EditR (https://moriaritylab.shinyapps.io/editr_v10/).

### DNA extraction and amplicon sequencing

Plant genomic DNA was extracted with the innuPREP Plant DNA Kit (Analytik Jena). The targeted sequences were amplified with specific primers, and the amplicons were purified with the GeneJET Gel Extraction Kit (Thermo Fisher Scientific) and quantified using Qubit™ 1X dsDNA High Sensitivity Kits (Thermo Fisher Scientific). Oligos used in this study are listed in Table S2. Equal amounts of PCR products were pooled and sequenced (GENEWIZ, AMPLICON‐EZ). Amplicon sequencing was performed three times for each target location using genomic DNA isolated from three different protoplast transformation experiments. The target sites in the sequenced reads were analysed for mutations using CRISPResso2 (crispresso2.pinellolab.org; Clement *et al*., 2019).

### Statistical analysis

All values are shown as means ± SEM (standard error of the mean). Statistical differences between the values were tested using two‐tailed unpaired Student's *t*‐tests by GraphPad (Prism; www.graphpad.com).

## Author contributions

D.Z. and J.B. designed the experiments. D.Z. performed the experiments and analysed the data. D.Z. and J.B. wrote the manuscript.

## Funding information

The work was conducted using university core funding.

## Supporting information


**Data S1** Aligned result of amplicon sequencing data sequences.

**Figure S1** Schematic illustration for assembling TALE‐ABEs.
**Figure S2** Schematic illustration for *Agrobacterium*‐mediated transient expression in tobacco leaves.
**Figure S3** Phenotypes of T_0_ rice plants.
**Figure S4** Genotypes of 12 T_0_ rice plants.
**Figure S5** Analysis of pTABE_v6 induced off‐target effects in the T_0_ rice plants.
**Table S1** Plasmids used in this study.
**Table S2** Oligos used in this study.
**Table S3** TALE binding sequences and corresponding RVDs.
**Method S1** TALE‐ABE user manual.

## Data Availability

The amplicon sequencing data have been deposited in an NCBI BioProject database (PRJNA909199). Plasmids used in this study will be made available through Addgene.
