# Supplementary material for: Development of TALE‐adenine base editors in plants
Source: Plant Biotechnol J. 2023 Nov 24;22(5):1067–77. doi: 10.1111/pbi.14246 (PMC11022790; doi:10.1111/pbi.14246)
Supplement: Supplementary file 2 — Figure S1 Schematic illustration for assembling TALE‐ABEs. Figure S2 Schematic illustration for Agrobacterium‐mediated transient expression in tobacco leaves. Figure S3 Phenotypes of T0 rice plants. Figure S4 Genotypes of 12 T0 rice plants. Figure S5 Analysis of pTABE_v6 induced off‐target effects in the T0 rice plants. Table S1 Plasmids used in this study. Table S2 Oligos used in this study. Table S3 TALE binding sequences and corresponding RVDs. Method S1 TALE‐ABE user manual. [file PBI-22-1067-s002.docx]

**Supplementary Information**

**Development of TALE adenine base editors in plants**

Dingbo Zhang, Jens Boch

Leibniz Universität Hannover, Institute of Plant Genetics, Herrenhäuser Str. 2, 30419 Hannover, Germany

[Supplemental figure 1. Schematic illustration for assembling TALE-ABEs. 3](#_Toc139282110)

[Supplemental figure 2. Schematic illustration for *Agrobacterium*-mediated transient expression in tobacco leaves. 4](#_Toc139282111)

[Supplemental figure 3. Phenotypes of T_0_ rice plants. 5](#_Toc139282112)

[Supplemental figure 4. Genotypes of 12 T_0_ rice plants. 7](#_Toc139282113)

[Supplemental figure 5. Analysis of pTABE_v6 induced off-target effects in the T_0_ rice plants. 9](#_Toc139282114)

[Supplemental table 1. Plasmids used in this study. 10](#_Toc139282115)

[Supplemental table 2. Oligos used in this study. 11](#_Toc139282116)

[Supplemental table 3. TALE binding sequences and corresponding RVDs. 12](#_Toc139282117)

[Supplemental methods 13](#_Toc139282118)

[Supplemental sequences. 16](#_Toc139282119)

Continued

**Supplemental Figure 1. Schematic illustration for assembling TALE-ABEs.**

**(A)** Golden-Gate assembly of TALE-ABEs based on the modular cloning (MoClo) design. According to the target sequence (with a thymine at the 5’ end), up to six single repeats are ligated into the corresponding receiving plasmids. Next, those multi-repeat modules are combined with promoter, NLS or MTS or CTP, N-terminal domain, C-terminal domain, Tad8e, DddA half, terminator (Ter) and level 1 acceptor vector to yield the level 1 TALE-ABE transcription unit. Four base overhang sequences are shown on the modules. NLS: Nuclear localization signal. MTS: Mitochondrial targeting signal. CTP: chloroplast transition peptide. **(B)** An example for cloning level 1 TABE. Level 1 vectors are using for transient transformation (protoplasts assay and infiltration of *N. benthamiana* leaves), level 2/M vectors are using for *Agrobacterium*-mediated stable plant transformation.

**Supplemental Figure 2. Schematic illustration for *Agrobacterium*-mediated transient expression in tobacco leaves.**

**(A)** Three *Agrobacterium tumefaciens* GV3101 strains containing level 1 TALE-ABEs and GUS reporter constructs respectively were mixed 1:1:1 and **(B)** inoculated into *N. benthamiana* leaves with a total OD600 of 0.8. **(C)** Two to three days after inoculation, **(D)** Two leaf discs (diameter 0.8 cm) were harvested from each inoculation spot. **(E)** Leaf tissues were homogenized with TissueLyses II (QIAGEN) and incubated with 4-methyl-umbelliferyl-β-D-glucuronide (MUG), **(F)** GUS activity was measured using TECAN reader and **(G)** the data were analysed.

**Supplemental Figure 3. Phenotypes of T_0_ rice plants.**

**(A)** Schematic of A•T-to-G•C editing in rice plants mediated by TALE-ABEs. A pair of pTABE_v6 plasmids, left pTABE_v6 and right pTABE_v6 are separately transformed into *A. tumefaciens* strain EHA105. Two *A. tumefaciens* strains, each containing one of the two pTABE_v6 plasmids, are mixed before transforming rice calli. The regenerated plants are genotyped after 6-7 weeks of selection on medium with 50 mg/L hygromycin. Hyg: Hygromycin. **(B)** Regenerated rice plants transformed with pTABE_v6 that targeting *OspsaA*. Phenotypes of seven representative transgenic lines grown in rooting medium. Bar = 1 cm.

Continue

**Supplemental Figure 4. Genotypes of 12 T_0_ rice plants.**

Sanger sequencing chromatograms from WT and 12 T_0_ rice plants are aligned to the reference *OspsaA* sequence, respectively. Left, right TALE binding sites and spacer regions are indicated in purple, mismatches are highlighted. WT: wild-type.

**
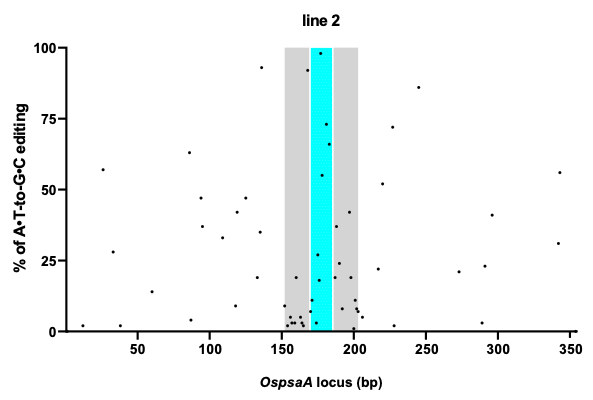
**
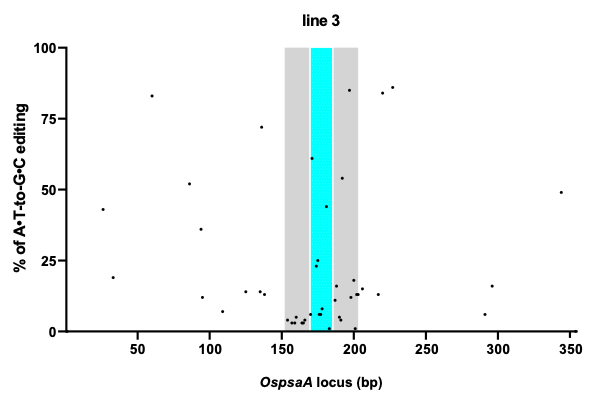


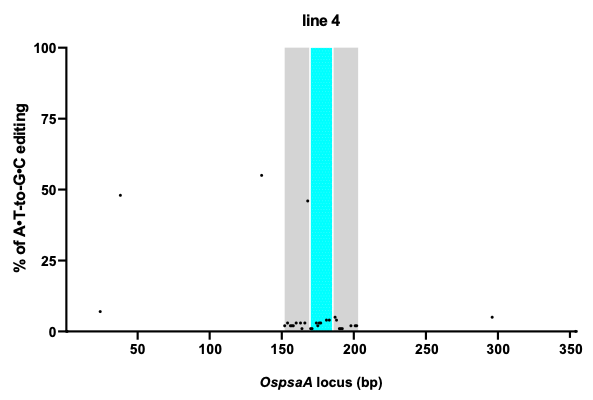

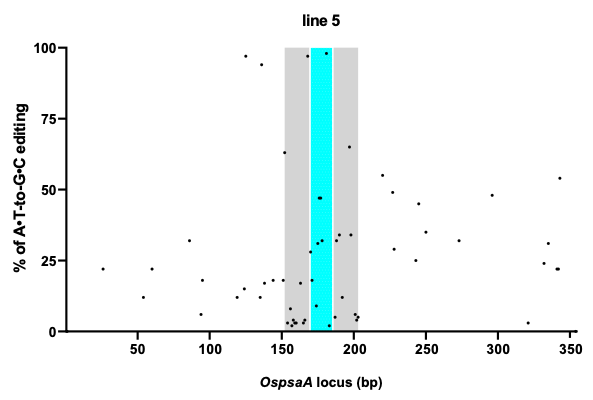


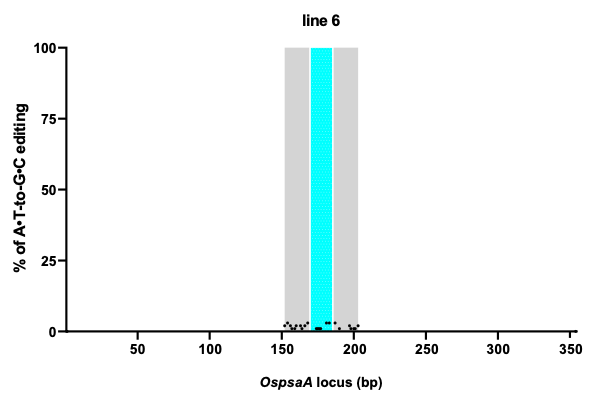

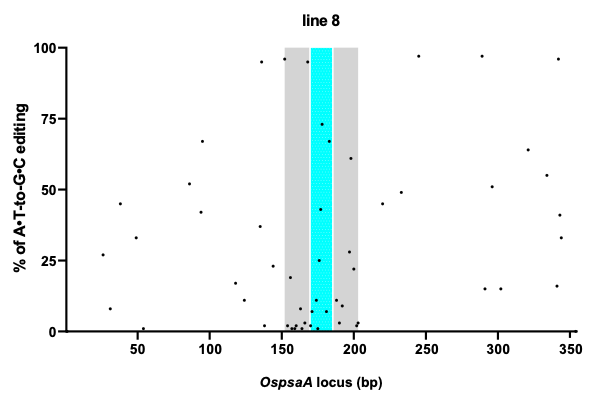


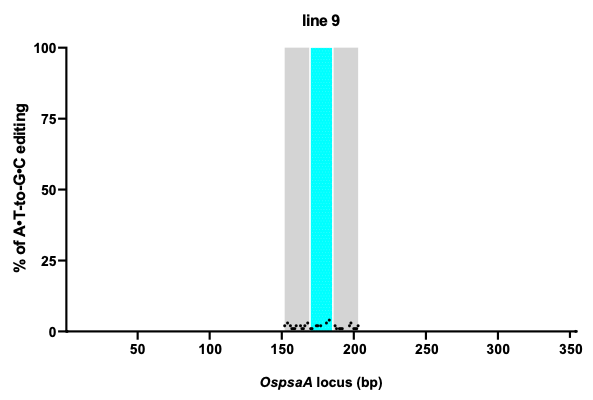

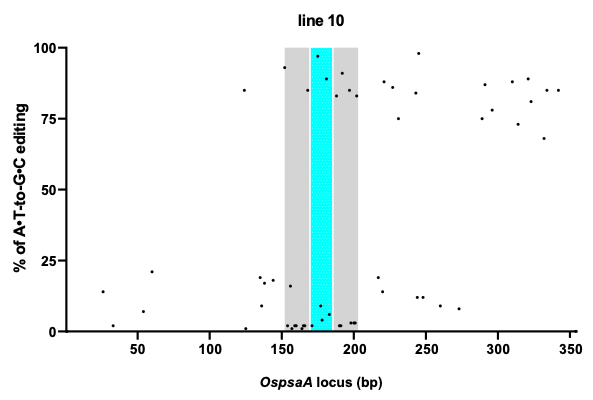


Continue

**
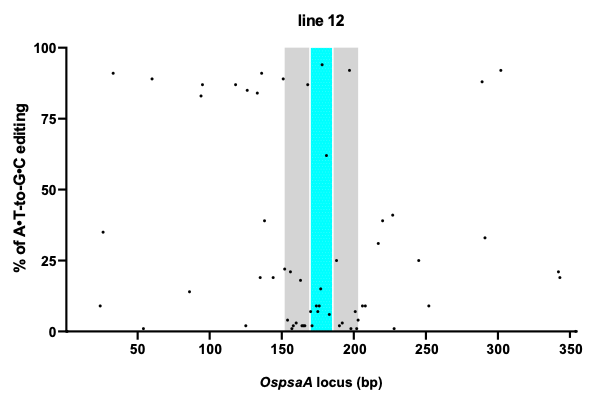
**

**Supplemental Figure 5.** **Analysis of pTABE_v6-induced off-target effects in the T_0_ rice plants.**

Dots showing the A•T-to-G•C editing frequencies relative to the reference sequence and the position ± 150 bp spanning the *OspsaA* target site. Left and right TALE binding sites are indicated in gray background, the spacer region is indicated in cyan background.

**Supplemental Table 1. Plasmids used in this study.**

| **Name** | **MoClo level** | **Content** |
| --- | --- | --- |
| L0_35s_Pro5U | level 0 | 2X35S promoter with GGAG/CCAT overhang |
| L0_ZmUbi_Pro5U | level 0 | Maize ubiquitin promoter with GGAG/CCAT overhang |
| L0_Noster | level 0 | Nos terminator with GCTT/AGGA overhang |
| L0_35s_ter | level 0 | 35S terminator with GCTT/AGGA overhang |
| L0_bpNLS | level 0 | bpNLS with CCAT/AATG overhang |
| L0_CTP | level 0 | Chloroplast transition peptide with CCAT/AATG overhang |
| L0_TND | level 0 | TALE N-Terminal domain (196 aa) with AATG/GAAC overhang |
| L0_TCD | level 0 | TALE C-Terminal domain (17 aa) with AGCA/TTCG overhang |
| L0_TadA8e_1 | level 0 | TadA8e with TTCG/AGTG overhang |
| L0_TadA8e_2 | level 0 | TadA8e with AGTG/GCTT overhang |
| L0_TadA8e_3 | level 0 | TadA8e with TTCG/GCTT overhang |
| L0_DddAE1347A_1 | level 0 | DddA^E1347A^ with AGTG/GCTT overhang |
| L0_DddAE1347A_2 | level 0 | DddA^E1347A^ with TTCG/GCTT overhang |
| L0_AID | level 0 | AID*Δ with AGTG/GCTT overhang |
| L0_Rad51DBD | level 0 | Rad51DBD with AGTG/GCTT overhang |
| L0_Sso7d | level 0 | Sso7d with AGTG/GCTT overhang |
| L0_DddA_N_1 | level 0 | DddA_N with TTCG/GCTT overhang |
| L0_DddA_N_2 | level 0 | DddA_N with AGTG/GCTT overhang |
| L0_DddA_C_1 | level 0 | DddA_C with TTCG/GCTT overhang |
| L0_DddA_C_2 | level 0 | DddA_C with AGTG/GCTT overhang |
| L0_DddA6_N | level 0 | DddA6_N with AGTG/GCTT overhang |
| L0_DddA6_C | level 0 | DddA6_C with AGTG/GCTT overhang |
| L0_DddA_N_UGI | level 0 | DddA_N_UGI with TTCG/GCTT overhang |
| L0_DddA_C_UGI | level 0 | DddA_C_UGI with AGTG/GCTT overhang |
| L0_NLS_A3AY130F | level 0 | A3A^Y130F^ with TTCG/TCAA overhang |
| L0_UGI | level 0 | UGI with TTCA/GCTT overhang |
| L1_Hyg_P3 | level 1 | Hygromycin expression unit in position 3 |
| pICH50892 | level 1 | End linker 3 for level M assembly |
| pICH47732_DZ | level 1 accepter | MoClo level 1 accepter in position 1 with GGAG/AGGA overhang |
| pICH47742_DZ | level 1 accepter | MoClo level 1 accepter in position 2 with GGAG/AGGA overhang |
| pICH47751_DZ | level 1 accepter | MoClo level 1 accepter in position 3 with GGAG/AGGA overhang |
| pICH47761_DZ | level 1 accepter | MoClo level 1 accepter in position 4 with GGAG/AGGA overhang |
| pGUS_WT | level M | Wild-type GUS expression plasmid containing a p19 silencing suppressor |
| pGUS_424 | level M | GUS^*424^ expression plasmid containing a p19 silencing suppressor |
| pGUS_537 | level M | GUS^G537^ expression plasmid containing a p19 silencing suppressor |
| pAGM8031 | level M accepter | MoClo level M accepter |
| For TALE repeats see Geiβler et al., 2011 | | |

**Supplemental Table 2. Oligos used in this study.**

| **Name** | **Sequence 5’ - 3’** | **Application** |
| --- | --- | --- |
| OsSweet14-TABE-AP-F1 | GAGATCAATGAGGCACAGTA | Amplify *OsSWEET14* |
| OsSweet14-AP-RE1-R1 | TGTCCAGGGTCACACACC |  |
| NB1-T1-AP-F1 | GATATTTATGTTTGTTTTGC | Amplify NB-T1 |
| NB1_R | CCTAGGACTCGAGAGAAAGCTGCAAATC |  |
| OsALS550-AP-F1 | TGCAGGAATATTGAACCC | Amplify Os*ALS* |
| OsALS550-AP-R1 | TATTGATGGGGATGGTAG |  |
| OsPDS-AP-F1 | CGTATCTGTATTCCGTAG | Amplify *OsPDS* |
| OsPDS-AP-R1 | ACCTCGTTTGCCTCCTC |  |
| GUS424-AP-F1 | TAACGGTTCAGGCACAGCAC | Amplify *GUS*  (Treatment with TadA8e-dSpCas9) |
| GUS424-AP-R1 | CAAGCCGAAAGAACTGTACA |  |
| GUS424-AP-F2 | CATTGACGCAGGTGATCGGA | Amplify *GUS*  (Treatment with pTABE_v6) |
| GUS424-AP- R1 | GATTGATGAAACTGCTGCT |  |
| PsA-F | GCTCCTGGTGCAACAACAAG | Amplify *OspsaA* |
| PsA-R | AGGCCCAGACAAAATGAGCA |  |

**Supplemental Table 3. TALE binding sequences and corresponding RVDs.**

| **Gene** |  | **Binding sequence ^†^**  **(5’-3’)** | **RVDs** |
| --- | --- | --- | --- |
| GUS^*424^ | left TALE1 | **TATTCCCGTGC** | **NI NG NG HD HD HD NN NG NN HD** |
|  | left TALE2 | **TATTCCCGTGCAC** | **NI NG NG HD HD HD NN NG NN HD NI HD** |
|  | left TALE3 | **TATTCCCGTGCACCT** | **NI NG NG HD HD HD NN NG NN HD NI HD HD NG** |
|  | right TALE1 | **TGCCAACGAACCGGA** | **NN HD HD NI NI HD NN NI NI HD HD NN NN NI** |
|  | right TALE2 | **TGCCAACGAACCGGATA** | **NN HD HD NI NI HD NN NI NI HD HD NN NN NI NG NI** |
|  | right TALE3 | **TGCCAACGAACCGGATACC** | **NN HD HD NI NI HD NN NI NI HD HD NN NN NI NG NI HD HD** |
|  | right TALE4 | **TGCCAACGAACCGGATACCCG** | **NN HD HD NI NI HD NN NI NI HD HD NN NN NI NG NI HD HD HD NN** |
|  | right TALE5 | **TGCCAACGAACCGGATACCCGTC** | **NN HD HD NI NI HD NN NI NI HD HD NN NN NI NG NI HD HD HD NN NG HD** |
| GUS^G537^ | left TALE | **TCAGCCGATTATCATC** | **HD NI NN HD HD NN NI NG NG NI NG HD NI NG HD** |
|  | right TALE | **TAACGTATCCACGCC** | **NI NI HD NN NG NI NG HD HD NI HD NN HD HD** |
| *OsALS* | left TALE | **TATGCGCCCTATTTGCCTT** | **NI NG NN HD NN HD HD HD NG NI NG NG NG NN HD HD NG NG** |
|  | right TALE | **TGGGTATGGTTGTGCAATG** | **NN NN NN NG NI NG NN NN NG NG NN NG NN HD NI NI NG NN** |
| *OsSWEET14* | left TALE | **TTGATGAGCTTAGCACCTG** | **NG NN NI NG NN NI NN HD NG NG NI NN HD NI HD HD NG NN** |
|  | right TALE | **TCTTCCTTCCTAGCACTAT** | **HD NG NG HD HD NG NG HD HD NG NI NN HD NI HD NG NI NG** |
| *OsPDS* | left TALE | **TTCCTGGTAAATAGGTAGT** | **NG HD HD NG NN NN NG NI NI NI NG NI NN NN NG NI NN NG** |
|  | right TALE | **TGCTCAAAGCCAGCAGTCA** | **NN HD NG HD NI NI NI NN HD HD NI NN HD NI NN NG HD NI** |
| *OspsaA* | left TALE | **TAGTCATTTTCCATTTCAG** | **NI NN NG HD NI NG NG NG NG HD HD NI NG NG NG HD NI NN** |
|  | right TALE | **TTATAGTACCCCAAACATC** | **NG HI NG NI NN NG NI HD HD HD HD NI NI NI HD NI NG HD** |
| NB-T1 | left TALE | **TAGCTTGTTCCACATT** | **NI NN HD NG NG NN NG NG HD**  **HD NI HD NI NG NG** |
|  | right TALE | **TCTGACCCAGCATGACA** | **HD NG NN NI HD HD HD NI NN HD NING NN NI HD NI** |

^†^: A red T indicates the base recognized by the N-terminal TALE domain.

**Supplemental Methods**

**TALE-ABE user manual**

The TALE-ABEs are designed for conversion of A•T base pairs to G•C base pairs in plant nuclear or organellar genomes. The plasmid cloning is based on the modular cloning (MoClo) system (Weber *et al*., 2011). BpiI and T4 DNA ligase from Thermo Fisher Scientific and BsaI-HF v2 from NEB are used in this protocol.

**Step1: TALE repeat assembly.**

- Up to six repeats can be installed into one assembly vector following the reaction below:

| component | volume |
| --- | --- |
| repeat 1 (50 ng/µl) | 1 µl |
| repeat 2 (50 ng/µl) | 1 µl |
| repeat 3 (50 ng/µl) | 1 µl |
| repeat 4 (50 ng/µl) | 1 µl |
| repeat 5 (50 ng/µl) | 1 µl |
| repeat 6 (50 ng/µl) | 1 µl |
| Assembly vector (50 ng/µl) | 1 µl |
| Buffer Green (Fisher Scientific) | 2 µl |
| 10 mM ATP | 2 µl |
| BpiI (Fisher Scientific) | 1 µl |
| T4 ligase (5 U/µl, Fisher Scientific) | 1 µl |
| H_2_O | Add to 20 µl |

Note: If less than six repeats are chosen, the last repeat should be used from the stop repeat library as shown in Supplemental Figure 1.

- Incubate reactions in a thermocycler with the following procedure:

| Temperature (°C) | Time (min) | Cycle |
| --- | --- | --- |
| 37 | 5 | 10 – 40x |
| 16 | 10 |  |
| 37 | 15 | - |
| 80 | 5 | - |
| 10 | Hold | - |

- Transform the reactions into *E. coli* and plate on LB medium with kanamycin and X-gal for blue-white screen. Confirm correct clones by digestion and Sanger sequencing.

**Step 2: level 1 assembly**

- Assemble the TALE-ABE level 1 transcription unit using the following Golden Gate reaction:

| component | volume |
| --- | --- |
| Cut Smart Buffer (NEB) | 2 µl |
| 10 mM ATP | 2 µl |
| level 1 acceptor vector (50 ng/µl) | 1 µl |
| level 0 module (promoter) (50 ng/µl) | 1 µl |
| level 0 module (NLS) (50 ng/µl) | 1 µl |
| level 0 module (N-terminal) (50 ng/µl) | 1 µl |
| level 0 module (TALE repeats from step1) | 1 µl |
| level 0 module (C-terminal) (50 ng/µl) | 1 µl |
| level 0 module (TadA8e) (50 ng/µl) | 1 µl |
| level 0 module (fusion domain) (50 ng/µl) | 1 µl |
| level 0 module (Terminator) (50 ng/µl) | 1 µl |
| BsaI-HF v2 (NEB) | 1 µl |
| T4 ligase (5 U/µl, Fisher Scientific) | 1 µl |
| H_2_O | Add to 20 µl |

- Incubate reactions in a thermocycler with the following procedure:

| Temperature (°C) | Time |
| --- | --- |
| 37 | 3 h – 12 h |
| 80 | 5 min |
| 10 | Hold |

- Transform the reactions into *E. coli* and plate on LB medium with ampicillin and X-gal for blue-white screen. Confirm correct clones by digestion.
- For transient expression in *N. benthamiana*, those correct plasmids are transformed into *Agrobacterium tumefaciens* strain GV3101 and plate on YEB medium with rifampin and carbenicillin. For protoplasts assay, isolate those correct plasmids with Midi prep kit (Qiagen).

**Step 3: level 2/M assembly**

- Assemble the binary level 2/M vectors as follows:

| component | volume |
| --- | --- |
| Green Buffer (Fisher Scientific) | 2 µl |
| 10 mM ATP | 2 µl |
| level 2/M acceptor vector (50 ng/µl) | 1 µl |
| level 1 module (TALE-ABE in position 1) (50 ng/µl) | 1 µl |
| level 1 module (TALE-ABE in position 2) (50 ng/µl) | 1 µl |
| level 1 module (L1_Hyg_P3 in position 3) (50 ng/µl) | 1 µl |
| level 1 module (pICH50892) (50 ng/µl) | 1 µl |
| BpiI (Fisher Scientific) | 1 µl |
| T4 ligase (5 U/µl, Fisher Scientific) | 1 µl |
| H_2_O | Add to 20 µl |

- Incubate reactions in a thermocycler with the following procedure:

| Temperature (°C) | Time (min) | Cycle |
| --- | --- | --- |
| 37 | 5 | 10 – 40x |
| 16 | 10 |  |
| 37 | 15 | - |
| 80 | 5 | - |
| 10 | Hold | - |

- Transform the reactions into *E. coli* and plate on LB medium with spectinomycin and X-gal for blue-white screen. Confirm correct clones by digestion.

**Supplemental Sequences.**

**Amino acid sequences of TALE-ABEs architectures.** One of each TALE-ABE (sTABE and pTABE) sequence is shown, RVDs are underline.

**Chloroplast transition peptide:**

MAPSVMASSATTVAPFQGLKSTAGMPVARRSGNSSFGNVSNGGRIRCMQVWPIEGIKKFETLSYLPPLGNSSFGNVSNGGRIRC

**sTABE_v1** (from N- to C-terminus):

bpNLS-N-terminal domain-**TALE repeats**-C-terminal domain-GS linker-TadA8e

MKRTADGSEFESPKKKRKVMAHHTEAATGEWDEVQSGLRAADAPPPTMRVAVTAARPPRAKPAPRRRAAQPSDASPAAQVDLRTLGYSQQQQEKIKPKVRSTVAQHHEALVGHGFTHAHIVALSQHPAALGTVAVKYQDMIAALPEATHEAIVGVGKQWSGARALEALLTVAGELRGPPLQLDTGQLLKIAKRGGVTAVEAVHAWRNALTGAPLN**LTPEQVVAIASNIGGKQALETVQRLLPVLCQAHGLTPEQVVAIASNGGGKQALETVQRLLPVLCQAHGLTPEQVVAIASNGGGKQALETVQRLLPVLCQAHGLTPEQVVAIASHDGGKQALETVQRLLPVLCQAHGLTPEQVVAIASHDGGKQALETVQRLLPVLCQAHGLTPEQVVAIASHDGGKQALETVQRLLPVLCQAHGLTPEQVVAIASNNGGKQALETVQRLLPVLCQAHGLTPEQVVAIASNGGGKQALETVQRLLPVLCQAHGLTPEQVVAIASNNGGKQALETVQRLLPVLCQAHGLTPEQVVAIASHDGGKQALETVQRLLPVLCQAHGLTPEQVVAIASNIGGKQALETVQRLLPVLCQAHGLTPEQVVAIASHDGGKQALE**SIVAQLSRPDPSLAALTGSSEVEFSHEYWMRHALTLAKRARDEREVPVGAVLVLNNRVIGEGWNRAIGLHDPTAHAEIMALRQGGLVMQNYRLIDATLYVTFEPCVMCAGAMIHSRIGRVVFGVRNSKRGAAGSLMNVLNYPGMNHRVEITEGILADECAALLCDFYRMPRQVFNAQKKAQSSIN*

**sTABE_v2** (from N- to C-terminus):

bpNLS-N-terminal domain-**TALE repeats**-C-terminal domain-GS linker-TadA8e-SGGS linker-TadA8e

MKRTADGSEFESPKKKRKVMAHHTEAATGEWDEVQSGLRAADAPPPTMRVAVTAARPPRAKPAPRRRAAQPSDASPAAQVDLRTLGYSQQQQEKIKPKVRSTVAQHHEALVGHGFTHAHIVALSQHPAALGTVAVKYQDMIAALPEATHEAIVGVGKQWSGARALEALLTVAGELRGPPLQLDTGQLLKIAKRGGVTAVEAVHAWRNALTGAPLN**LTPEQVVAIASNIGGKQALETVQRLLPVLCQAHGLTPEQVVAIASNGGGKQALETVQRLLPVLCQAHGLTPEQVVAIASNGGGKQALETVQRLLPVLCQAHGLTPEQVVAIASHDGGKQALETVQRLLPVLCQAHGLTPEQVVAIASHDGGKQALETVQRLLPVLCQAHGLTPEQVVAIASHDGGKQALETVQRLLPVLCQAHGLTPEQVVAIASNNGGKQALETVQRLLPVLCQAHGLTPEQVVAIASNGGGKQALETVQRLLPVLCQAHGLTPEQVVAIASNNGGKQALETVQRLLPVLCQAHGLTPEQVVAIASHDGGKQALETVQRLLPVLCQAHGLTPEQVVAIASNIGGKQALETVQRLLPVLCQAHGLTPEQVVAIASHDGGKQALE**SIVAQLSRPDPSLAALTGSSEVEFSHEYWMRHALTLAKRARDEREVPVGAVLVLNNRVIGEGWNRAIGLHDPTAHAEIMALRQGGLVMQNYRLIDATLYVTFEPCVMCAGAMIHSRIGRVVFGVRNSKRGAAGSLMNVLNYPGMNHRVEITEGILADECAALLCDFYRMPRQVFNAQKKAQSSINSGGSSEVEFSHEYWMRHALTLAKRARDEREVPVGAVLVLNNRVIGEGWNRAIGLHDPTAHAEIMALRQGGLVMQNYRLIDATLYVTFEPCVMCAGAMIHSRIGRVVFGVRNSKRGAAGSLMNVLNYPGMNHRVEITEGILADECAALLCDFYRMPRQVFNAQKKAQSSIN*

**sTABE_v3** (from N- to C-terminus):

bpNLS-N-terminal domain-**TALE repeats**-C-terminal domain-GS linker-TadA8e-SGGS linker-DddA^E1347A^

MKRTADGSEFESPKKKRKVMAHHTEAATGEWDEVQSGLRAADAPPPTMRVAVTAARPPRAKPAPRRRAAQPSDASPAAQVDLRTLGYSQQQQEKIKPKVRSTVAQHHEALVGHGFTHAHIVALSQHPAALGTVAVKYQDMIAALPEATHEAIVGVGKQWSGARALEALLTVAGELRGPPLQLDTGQLLKIAKRGGVTAVEAVHAWRNALTGAPLN**LTPEQVVAIASNIGGKQALETVQRLLPVLCQAHGLTPEQVVAIASNGGGKQALETVQRLLPVLCQAHGLTPEQVVAIASNGGGKQALETVQRLLPVLCQAHGLTPEQVVAIASHDGGKQALETVQRLLPVLCQAHGLTPEQVVAIASHDGGKQALETVQRLLPVLCQAHGLTPEQVVAIASHDGGKQALETVQRLLPVLCQAHGLTPEQVVAIASNNGGKQALETVQRLLPVLCQAHGLTPEQVVAIASNGGGKQALETVQRLLPVLCQAHGLTPEQVVAIASNNGGKQALETVQRLLPVLCQAHGLTPEQVVAIASHDGGKQALETVQRLLPVLCQAHGLTPEQVVAIASNIGGKQALETVQRLLPVLCQAHGLTPEQVVAIASHDGGKQALE**SIVAQLSRPDPSLAALTGSSEVEFSHEYWMRHALTLAKRARDEREVPVGAVLVLNNRVIGEGWNRAIGLHDPTAHAEIMALRQGGLVMQNYRLIDATLYVTFEPCVMCAGAMIHSRIGRVVFGVRNSKRGAAGSLMNVLNYPGMNHRVEITEGILADECAALLCDFYRMPRQVFNAQKKAQSSINSGGSGSYALGPYQISAPQLPAYNGQTVGTFYYVNDAGGLESKVFSSGGPTPYPNYANAGHVAGQSALFMRDNGISEGLVFHNNPEGTCGFCVNMTETLLPENAKMTVVPPEGAIPVKRGATGETKVFTGNSNSPKSPTKGGC*

**sTABE_v4** (from N- to C-terminus):

bpNLS-N-terminal domain-**TALE repeats**-C-terminal domain-GS linker-TadA8e-SGGS linker- AID*Δ

MKRTADGSEFESPKKKRKVMAHHTEAATGEWDEVQSGLRAADAPPPTMRVAVTAARPPRAKPAPRRRAAQPSDASPAAQVDLRTLGYSQQQQEKIKPKVRSTVAQHHEALVGHGFTHAHIVALSQHPAALGTVAVKYQDMIAALPEATHEAIVGVGKQWSGARALEALLTVAGELRGPPLQLDTGQLLKIAKRGGVTAVEAVHAWRNALTGAPLN**LTPEQVVAIASNIGGKQALETVQRLLPVLCQAHGLTPEQVVAIASNGGGKQALETVQRLLPVLCQAHGLTPEQVVAIASNGGGKQALETVQRLLPVLCQAHGLTPEQVVAIASHDGGKQALETVQRLLPVLCQAHGLTPEQVVAIASHDGGKQALETVQRLLPVLCQAHGLTPEQVVAIASHDGGKQALETVQRLLPVLCQAHGLTPEQVVAIASNNGGKQALETVQRLLPVLCQAHGLTPEQVVAIASNGGGKQALETVQRLLPVLCQAHGLTPEQVVAIASNNGGKQALETVQRLLPVLCQAHGLTPEQVVAIASHDGGKQALETVQRLLPVLCQAHGLTPEQVVAIASNIGGKQALETVQRLLPVLCQAHGLTPEQVVAIASHDGGKQALE**SIVAQLSRPDPSLAALTGSSEVEFSHEYWMRHALTLAKRARDEREVPVGAVLVLNNRVIGEGWNRAIGLHDPTAHAEIMALRQGGLVMQNYRLIDATLYVTFEPCVMCAGAMIHSRIGRVVFGVRNSKRGAAGSLMNVLNYPGMNHRVEITEGILADECAALLCDFYRMPRQVFNAQKKAQSSINSGGSMDSLLMNRREFLYQFKNVRWAKGRRETYLCYVVKRRDSATSFSLDFGYLRNKNGCHVELLFLRYISDWDLDPGRCYRVTWFISWSPCYDCARHVADFLRGNPNLSLRIFTARLYFCEDRKAEPEGLRRLHRAGVQIAIMTFKDYFYCWNTFVENHGRTFKAWEGLHENSVRLSRQLRRILLPLYEVDDLRDAFRT*

**sTABE_v5** (from N- to C-terminus):

bpNLS-N-terminal domain-**TALE repeats**-C-terminal domain-GS linker-TadA8e-SGGS linker- Rad51DBD

MKRTADGSEFESPKKKRKVMAHHTEAATGEWDEVQSGLRAADAPPPTMRVAVTAARPPRAKPAPRRRAAQPSDASPAAQVDLRTLGYSQQQQEKIKPKVRSTVAQHHEALVGHGFTHAHIVALSQHPAALGTVAVKYQDMIAALPEATHEAIVGVGKQWSGARALEALLTVAGELRGPPLQLDTGQLLKIAKRGGVTAVEAVHAWRNALTGAPLN**LTPEQVVAIASNIGGKQALETVQRLLPVLCQAHGLTPEQVVAIASNGGGKQALETVQRLLPVLCQAHGLTPEQVVAIASNGGGKQALETVQRLLPVLCQAHGLTPEQVVAIASHDGGKQALETVQRLLPVLCQAHGLTPEQVVAIASHDGGKQALETVQRLLPVLCQAHGLTPEQVVAIASHDGGKQALETVQRLLPVLCQAHGLTPEQVVAIASNNGGKQALETVQRLLPVLCQAHGLTPEQVVAIASNGGGKQALETVQRLLPVLCQAHGLTPEQVVAIASNNGGKQALETVQRLLPVLCQAHGLTPEQVVAIASHDGGKQALETVQRLLPVLCQAHGLTPEQVVAIASNIGGKQALETVQRLLPVLCQAHGLTPEQVVAIASHDGGKQALE**SIVAQLSRPDPSLAALTGSSEVEFSHEYWMRHALTLAKRARDEREVPVGAVLVLNNRVIGEGWNRAIGLHDPTAHAEIMALRQGGLVMQNYRLIDATLYVTFEPCVMCAGAMIHSRIGRVVFGVRNSKRGAAGSLMNVLNYPGMNHRVEITEGILADECAALLCDFYRMPRQVFNAQKKAQSSINSGGSAMQMQLEANADTSVEEESFGPQPISRLEQCGINANDVKKLEEAGFHTVEAVAYAPKKELINIKGISEAKADKILAEAAKLVPMGFTTATEFHQRRSEIIQITTGSKELDKLLQ*

**sTABE_v6** (from N- to C-terminus):

bpNLS-N-terminal domain-**TALE repeats**-C-terminal domain-GS linker-TadA8e-SGGS linker- Sso7d

MKRTADGSEFESPKKKRKVMAHHTEAATGEWDEVQSGLRAADAPPPTMRVAVTAARPPRAKPAPRRRAAQPSDASPAAQVDLRTLGYSQQQQEKIKPKVRSTVAQHHEALVGHGFTHAHIVALSQHPAALGTVAVKYQDMIAALPEATHEAIVGVGKQWSGARALEALLTVAGELRGPPLQLDTGQLLKIAKRGGVTAVEAVHAWRNALTGAPLN**LTPEQVVAIASNIGGKQALETVQRLLPVLCQAHGLTPEQVVAIASNGGGKQALETVQRLLPVLCQAHGLTPEQVVAIASNGGGKQALETVQRLLPVLCQAHGLTPEQVVAIASHDGGKQALETVQRLLPVLCQAHGLTPEQVVAIASHDGGKQALETVQRLLPVLCQAHGLTPEQVVAIASHDGGKQALETVQRLLPVLCQAHGLTPEQVVAIASNNGGKQALETVQRLLPVLCQAHGLTPEQVVAIASNGGGKQALETVQRLLPVLCQAHGLTPEQVVAIASNNGGKQALETVQRLLPVLCQAHGLTPEQVVAIASHDGGKQALETVQRLLPVLCQAHGLTPEQVVAIASNIGGKQALETVQRLLPVLCQAHGLTPEQVVAIASHDGGKQALE**SIVAQLSRPDPSLAALTGSSEVEFSHEYWMRHALTLAKRARDEREVPVGAVLVLNNRVIGEGWNRAIGLHDPTAHAEIMALRQGGLVMQNYRLIDATLYVTFEPCVMCAGAMIHSRIGRVVFGVRNSKRGAAGSLMNVLNYPGMNHRVEITEGILADECAALLCDFYRMPRQVFNAQKKAQSSINSGGSATVKFKYKGEEKEVDISKIKKVWRVGKMISFTYDEGGGKTGRGAVSEKDAPKELLQMLEKQKKG*

**pTABE_v1** (from N- to C-terminus):

bpNLS-N-terminal domain-**TALE repeats**-C-terminal domain-GS linker-TadA8e

bpNLS-N-terminal domain-**TALE repeats**-C-terminal domain-GSSGGS linker-DddA^E1347A^

MKRTADGSEFESPKKKRKVMAHHTEAATGEWDEVQSGLRAADAPPPTMRVAVTAARPPRAKPAPRRRAAQPSDASPAAQVDLRTLGYSQQQQEKIKPKVRSTVAQHHEALVGHGFTHAHIVALSQHPAALGTVAVKYQDMIAALPEATHEAIVGVGKQWSGARALEALLTVAGELRGPPLQLDTGQLLKIAKRGGVTAVEAVHAWRNALTGAPLN**LTPEQVVAIASNIGGKQALETVQRLLPVLCQAHGLTPEQVVAIASNGGGKQALETVQRLLPVLCQAHGLTPEQVVAIASNGGGKQALETVQRLLPVLCQAHGLTPEQVVAIASHDGGKQALETVQRLLPVLCQAHGLTPEQVVAIASHDGGKQALETVQRLLPVLCQAHGLTPEQVVAIASHDGGKQALETVQRLLPVLCQAHGLTPEQVVAIASNNGGKQALETVQRLLPVLCQAHGLTPEQVVAIASNGGGKQALETVQRLLPVLCQAHGLTPEQVVAIASNNGGKQALETVQRLLPVLCQAHGLTPEQVVAIASHDGGKQALETVQRLLPVLCQAHGLTPEQVVAIASNIGGKQALETVQRLLPVLCQAHGLTPEQVVAIASHDGGKQALE**SIVAQLSRPDPSLAALTGSSEVEFSHEYWMRHALTLAKRARDEREVPVGAVLVLNNRVIGEGWNRAIGLHDPTAHAEIMALRQGGLVMQNYRLIDATLYVTFEPCVMCAGAMIHSRIGRVVFGVRNSKRGAAGSLMNVLNYPGMNHRVEITEGILADECAALLCDFYRMPRQVFNAQKKAQSSIN*

MKRTADGSEFESPKKKRKVMAHHTEAATGEWDEVQSGLRAADAPPPTMRVAVTAARPPRAKPAPRRRAAQPSDASPAAQVDLRTLGYSQQQQEKIKPKVRSTVAQHHEALVGHGFTHAHIVALSQHPAALGTVAVKYQDMIAALPEATHEAIVGVGKQWSGARALEALLTVAGELRGPPLQLDTGQLLKIAKRGGVTAVEAVHAWRNALTGAPLN**LTPEQVVAIASNNGGKQALETVQRLLPVLCQAHGLTPEQVVAIASHDGGKQALETVQRLLPVLCQAHGLTPEQVVAIASHDGGKQALETVQRLLPVLCQAHGLTPEQVVAIASNIGGKQALETVQRLLPVLCQAHGLTPEQVVAIASNIGGKQALETVQRLLPVLCQAHGLTPEQVVAIASHDGGKQALETVQRLLPVLCQAHGLTPEQVVAIASNNGGKQALETVQRLLPVLCQAHGLTPEQVVAIASNIGGKQALETVQRLLPVLCQAHGLTPEQVVAIASNIGGKQALETVQRLLPVLCQAHGLTPEQVVAIASHDGGKQALETVQRLLPVLCQAHGLTPEQVVAIASHDGGKQALETVQRLLPVLCQAHGLTPEQVVAIASNNGGKQALETVQRLLPVLCQAHGLTPEQVVAIASNNGGKQALETVQRLLPVLCQAHGLTPEQVVAIASNIGGKQALETVQRLLPVLCQAHGLTPEQVVAIASNGGGKQALETVQRLLPVLCQAHGLTPEQVVAIASNIGGKQALE**SIVAQLSRPDPSLAALTGSSGGSGSYALGPYQISAPQLPAYNGQTVGTFYYVNDAGGLESKVFSSGGPTPYPNYANAGHVAGQSALFMRDNGISEGLVFHNNPEGTCGFCVNMTETLLPENAKMTVVPPEGAIPVKRGATGETKVFTGNSNSPKSPTKGGC*

**pTABE_v2** (from N- to C-terminus):

bpNLS-N-terminal domain-**TALE repeats**-C-terminal domain-GS linker-TadA8e-SGGS linker-DddA C half

bpNLS-N-terminal domain-**TALE repeats**-C-terminal domain-GS linker-DddA N half

MKRTADGSEFESPKKKRKVMAHHTEAATGEWDEVQSGLRAADAPPPTMRVAVTAARPPRAKPAPRRRAAQPSDASPAAQVDLRTLGYSQQQQEKIKPKVRSTVAQHHEALVGHGFTHAHIVALSQHPAALGTVAVKYQDMIAALPEATHEAIVGVGKQWSGARALEALLTVAGELRGPPLQLDTGQLLKIAKRGGVTAVEAVHAWRNALTGAPLN**LTPEQVVAIASNNGGKQALETVQRLLPVLCQAHGLTPEQVVAIASHDGGKQALETVQRLLPVLCQAHGLTPEQVVAIASHDGGKQALETVQRLLPVLCQAHGLTPEQVVAIASNIGGKQALETVQRLLPVLCQAHGLTPEQVVAIASNIGGKQALETVQRLLPVLCQAHGLTPEQVVAIASHDGGKQALETVQRLLPVLCQAHGLTPEQVVAIASNNGGKQALETVQRLLPVLCQAHGLTPEQVVAIASNIGGKQALETVQRLLPVLCQAHGLTPEQVVAIASNIGGKQALETVQRLLPVLCQAHGLTPEQVVAIASHDGGKQALETVQRLLPVLCQAHGLTPEQVVAIASHDGGKQALETVQRLLPVLCQAHGLTPEQVVAIASNNGGKQALETVQRLLPVLCQAHGLTPEQVVAIASNNGGKQALETVQRLLPVLCQAHGLTPEQVVAIASNIGGKQALETVQRLLPVLCQAHGLTPEQVVAIASNGGGKQALETVQRLLPVLCQAHGLTPEQVVAIASNIGGKQALE**SIVAQLSRPDPSLAALTGSSEVEFSHEYWMRHALTLAKRARDEREVPVGAVLVLNNRVIGEGWNRAIGLHDPTAHAEIMALRQGGLVMQNYRLIDATLYVTFEPCVMCAGAMIHSRIGRVVFGVRNSKRGAAGSLMNVLNYPGMNHRVEITEGILADECAALLCDFYRMPRQVFNAQKKAQSSINSGGSAIPVKRGATGETKVFTGNSNSPKSPTKGGC*

MKRTADGSEFESPKKKRKVMAHHTEAATGEWDEVQSGLRAADAPPPTMRVAVTAARPPRAKPAPRRRAAQPSDASPAAQVDLRTLGYSQQQQEKIKPKVRSTVAQHHEALVGHGFTHAHIVALSQHPAALGTVAVKYQDMIAALPEATHEAIVGVGKQWSGARALEALLTVAGELRGPPLQLDTGQLLKIAKRGGVTAVEAVHAWRNALTGAPLN**LTPEQVVAIASNIGGKQALETVQRLLPVLCQAHGLTPEQVVAIASNGGGKQALETVQRLLPVLCQAHGLTPEQVVAIASNGGGKQALETVQRLLPVLCQAHGLTPEQVVAIASHDGGKQALETVQRLLPVLCQAHGLTPEQVVAIASHDGGKQALETVQRLLPVLCQAHGLTPEQVVAIASHDGGKQALETVQRLLPVLCQAHGLTPEQVVAIASNNGGKQALETVQRLLPVLCQAHGLTPEQVVAIASNGGGKQALETVQRLLPVLCQAHGLTPEQVVAIASNNGGKQALETVQRLLPVLCQAHGLTPEQVVAIASHDGGKQALETVQRLLPVLCQAHGLTPEQVVAIASNIGGKQALETVQRLLPVLCQAHGLTPEQVVAIASHDGGKQALE**SIVAQLSRPDPSLAALTGSGSYALGPYQISAPQLPAYNGQTVGTFYYVNDAGGLESKVFSSGGPTPYPNYANAGHVEGQSALFMRDNGISEGLVFHNNPEGTCGFCVNMTETLLPENAKMTVVPPEG*

**pTABE_v3** (from N- to C-terminus):

bpNLS-N-terminal domain-**TALE repeats**-C-terminal domain-GSGGS linker-DddA C half

bpNLS-N-terminal domain-**TALE repeats**-C-terminal domain-GS linker-TadA8e-SG linker-DddA N half

MKRTADGSEFESPKKKRKVMAHHTEAATGEWDEVQSGLRAADAPPPTMRVAVTAARPPRAKPAPRRRAAQPSDASPAAQVDLRTLGYSQQQQEKIKPKVRSTVAQHHEALVGHGFTHAHIVALSQHPAALGTVAVKYQDMIAALPEATHEAIVGVGKQWSGARALEALLTVAGELRGPPLQLDTGQLLKIAKRGGVTAVEAVHAWRNALTGAPLN**LTPEQVVAIASNNGGKQALETVQRLLPVLCQAHGLTPEQVVAIASHDGGKQALETVQRLLPVLCQAHGLTPEQVVAIASHDGGKQALETVQRLLPVLCQAHGLTPEQVVAIASNIGGKQALETVQRLLPVLCQAHGLTPEQVVAIASNIGGKQALETVQRLLPVLCQAHGLTPEQVVAIASHDGGKQALETVQRLLPVLCQAHGLTPEQVVAIASNNGGKQALETVQRLLPVLCQAHGLTPEQVVAIASNIGGKQALETVQRLLPVLCQAHGLTPEQVVAIASNIGGKQALETVQRLLPVLCQAHGLTPEQVVAIASHDGGKQALETVQRLLPVLCQAHGLTPEQVVAIASHDGGKQALETVQRLLPVLCQAHGLTPEQVVAIASNNGGKQALETVQRLLPVLCQAHGLTPEQVVAIASNNGGKQALETVQRLLPVLCQAHGLTPEQVVAIASNIGGKQALETVQRLLPVLCQAHGLTPEQVVAIASNGGGKQALETVQRLLPVLCQAHGLTPEQVVAIASNIGGKQALE**SIVAQLSRPDPSLAALTGSGGSAIPVKRGATGETKVFTGNSNSPKSPTKGGC*

MKRTADGSEFESPKKKRKVMAHHTEAATGEWDEVQSGLRAADAPPPTMRVAVTAARPPRAKPAPRRRAAQPSDASPAAQVDLRTLGYSQQQQEKIKPKVRSTVAQHHEALVGHGFTHAHIVALSQHPAALGTVAVKYQDMIAALPEATHEAIVGVGKQWSGARALEALLTVAGELRGPPLQLDTGQLLKIAKRGGVTAVEAVHAWRNALTGAPLN**LTPEQVVAIASNIGGKQALETVQRLLPVLCQAHGLTPEQVVAIASNGGGKQALETVQRLLPVLCQAHGLTPEQVVAIASNGGGKQALETVQRLLPVLCQAHGLTPEQVVAIASHDGGKQALETVQRLLPVLCQAHGLTPEQVVAIASHDGGKQALETVQRLLPVLCQAHGLTPEQVVAIASHDGGKQALETVQRLLPVLCQAHGLTPEQVVAIASNNGGKQALETVQRLLPVLCQAHGLTPEQVVAIASNGGGKQALETVQRLLPVLCQAHGLTPEQVVAIASNNGGKQALETVQRLLPVLCQAHGLTPEQVVAIASHDGGKQALETVQRLLPVLCQAHGLTPEQVVAIASNIGGKQALETVQRLLPVLCQAHGLTPEQVVAIASHDGGKQALE**SIVAQLSRPDPSLAALTGSSEVEFSHEYWMRHALTLAKRARDEREVPVGAVLVLNNRVIGEGWNRAIGLHDPTAHAEIMALRQGGLVMQNYRLIDATLYVTFEPCVMCAGAMIHSRIGRVVFGVRNSKRGAAGSLMNVLNYPGMNHRVEITEGILADECAALLCDFYRMPRQVFNAQKKAQSSINSGGSYALGPYQISAPQLPAYNGQTVGTFYYVNDAGGLESKVFSSGGPTPYPNYANAGHVEGQSALFMRDNGISEGLVFHNNPEGTCGFCVNMTETLLPENAKMTVVPPEG*

**pTABE_v4** (from N- to C-terminus):

bpNLS-N-terminal domain-**TALE repeats**-C-terminal domain-GS linker-TadA8e-SGGS linker-DddA C half-SGGS linker-UGI-SGGS linker-NLS

bpNLS-N-terminal domain-**TALE repeats**-C-terminal domain-GS linker-DddA N half-SGGS linker-UGI-SGGS linker-NLS

MKRTADGSEFESPKKKRKVMAHHTEAATGEWDEVQSGLRAADAPPPTMRVAVTAARPPRAKPAPRRRAAQPSDASPAAQVDLRTLGYSQQQQEKIKPKVRSTVAQHHEALVGHGFTHAHIVALSQHPAALGTVAVKYQDMIAALPEATHEAIVGVGKQWSGARALEALLTVAGELRGPPLQLDTGQLLKIAKRGGVTAVEAVHAWRNALTGAPLN**LTPEQVVAIASNNGGKQALETVQRLLPVLCQAHGLTPEQVVAIASHDGGKQALETVQRLLPVLCQAHGLTPEQVVAIASHDGGKQALETVQRLLPVLCQAHGLTPEQVVAIASNIGGKQALETVQRLLPVLCQAHGLTPEQVVAIASNIGGKQALETVQRLLPVLCQAHGLTPEQVVAIASHDGGKQALETVQRLLPVLCQAHGLTPEQVVAIASNNGGKQALETVQRLLPVLCQAHGLTPEQVVAIASNIGGKQALETVQRLLPVLCQAHGLTPEQVVAIASNIGGKQALETVQRLLPVLCQAHGLTPEQVVAIASHDGGKQALETVQRLLPVLCQAHGLTPEQVVAIASHDGGKQALETVQRLLPVLCQAHGLTPEQVVAIASNNGGKQALETVQRLLPVLCQAHGLTPEQVVAIASNNGGKQALETVQRLLPVLCQAHGLTPEQVVAIASNIGGKQALETVQRLLPVLCQAHGLTPEQVVAIASNGGGKQALETVQRLLPVLCQAHGLTPEQVVAIASNIGGKQALE**SIVAQLSRPDPSLAALTGSSEVEFSHEYWMRHALTLAKRARDEREVPVGAVLVLNNRVIGEGWNRAIGLHDPTAHAEIMALRQGGLVMQNYRLIDATLYVTFEPCVMCAGAMIHSRIGRVVFGVRNSKRGAAGSLMNVLNYPGMNHRVEITEGILADECAALLCDFYRMPRQVFNAQKKAQSSINSGGSAIPVKRGATGETKVFTGNSNSPKSPTKGGCSGGSTNLSDIIEKETGKQLVIQESILMLPEEVEEVIGNKPESDILVHTAYDESTDENVMLLTSDAPEYKPWALVIQDSNGENKIKMLSGGSPKKKRKV*

MKRTADGSEFESPKKKRKVMAHHTEAATGEWDEVQSGLRAADAPPPTMRVAVTAARPPRAKPAPRRRAAQPSDASPAAQVDLRTLGYSQQQQEKIKPKVRSTVAQHHEALVGHGFTHAHIVALSQHPAALGTVAVKYQDMIAALPEATHEAIVGVGKQWSGARALEALLTVAGELRGPPLQLDTGQLLKIAKRGGVTAVEAVHAWRNALTGAPLN**LTPEQVVAIASNIGGKQALETVQRLLPVLCQAHGLTPEQVVAIASNGGGKQALETVQRLLPVLCQAHGLTPEQVVAIASNGGGKQALETVQRLLPVLCQAHGLTPEQVVAIASHDGGKQALETVQRLLPVLCQAHGLTPEQVVAIASHDGGKQALETVQRLLPVLCQAHGLTPEQVVAIASHDGGKQALETVQRLLPVLCQAHGLTPEQVVAIASNNGGKQALETVQRLLPVLCQAHGLTPEQVVAIASNGGGKQALETVQRLLPVLCQAHGLTPEQVVAIASNNGGKQALETVQRLLPVLCQAHGLTPEQVVAIASHDGGKQALETVQRLLPVLCQAHGLTPEQVVAIASNIGGKQALETVQRLLPVLCQAHGLTPEQVVAIASHDGGKQALE**SIVAQLSRPDPSLAALTGSGSYALGPYQISAPQLPAYNGQTVGTFYYVNDAGGLESKVFSSGGPTPYPNYANAGHVEGQSALFMRDNGISEGLVFHNNPEGTCGFCVNMTETLLPENAKMTVVPPEGSGGSTNLSDIIEKETGKQLVIQESILMLPEEVEEVIGNKPESDILVHTAYDESTDENVMLLTSDAPEYKPWALVIQDSNGENKIKMLSGGSPKKKRKV*

**pTABE_v5** (from N- to C-terminus):

bpNLS-N-terminal domain-**TALE repeats**-C-terminal domain-GS linker-TadA8e-SGGS linker-DddA C half

bpNLS-N-terminal domain-**TALE repeats**-C-terminal domain-GS linker-TadA8e-SG linker-DddA N half

MKRTADGSEFESPKKKRKVMAHHTEAATGEWDEVQSGLRAADAPPPTMRVAVTAARPPRAKPAPRRRAAQPSDASPAAQVDLRTLGYSQQQQEKIKPKVRSTVAQHHEALVGHGFTHAHIVALSQHPAALGTVAVKYQDMIAALPEATHEAIVGVGKQWSGARALEALLTVAGELRGPPLQLDTGQLLKIAKRGGVTAVEAVHAWRNALTGAPLN**LTPEQVVAIASNNGGKQALETVQRLLPVLCQAHGLTPEQVVAIASHDGGKQALETVQRLLPVLCQAHGLTPEQVVAIASHDGGKQALETVQRLLPVLCQAHGLTPEQVVAIASNIGGKQALETVQRLLPVLCQAHGLTPEQVVAIASNIGGKQALETVQRLLPVLCQAHGLTPEQVVAIASHDGGKQALETVQRLLPVLCQAHGLTPEQVVAIASNNGGKQALETVQRLLPVLCQAHGLTPEQVVAIASNIGGKQALETVQRLLPVLCQAHGLTPEQVVAIASNIGGKQALETVQRLLPVLCQAHGLTPEQVVAIASHDGGKQALETVQRLLPVLCQAHGLTPEQVVAIASHDGGKQALETVQRLLPVLCQAHGLTPEQVVAIASNNGGKQALETVQRLLPVLCQAHGLTPEQVVAIASNNGGKQALETVQRLLPVLCQAHGLTPEQVVAIASNIGGKQALETVQRLLPVLCQAHGLTPEQVVAIASNGGGKQALETVQRLLPVLCQAHGLTPEQVVAIASNIGGKQALE**SIVAQLSRPDPSLAALTGSSEVEFSHEYWMRHALTLAKRARDEREVPVGAVLVLNNRVIGEGWNRAIGLHDPTAHAEIMALRQGGLVMQNYRLIDATLYVTFEPCVMCAGAMIHSRIGRVVFGVRNSKRGAAGSLMNVLNYPGMNHRVEITEGILADECAALLCDFYRMPRQVFNAQKKAQSSINSGGSAIPVKRGATGETKVFTGNSNSPKSPTKGGC*

MKRTADGSEFESPKKKRKVMAHHTEAATGEWDEVQSGLRAADAPPPTMRVAVTAARPPRAKPAPRRRAAQPSDASPAAQVDLRTLGYSQQQQEKIKPKVRSTVAQHHEALVGHGFTHAHIVALSQHPAALGTVAVKYQDMIAALPEATHEAIVGVGKQWSGARALEALLTVAGELRGPPLQLDTGQLLKIAKRGGVTAVEAVHAWRNALTGAPLN**LTPEQVVAIASNIGGKQALETVQRLLPVLCQAHGLTPEQVVAIASNGGGKQALETVQRLLPVLCQAHGLTPEQVVAIASNGGGKQALETVQRLLPVLCQAHGLTPEQVVAIASHDGGKQALETVQRLLPVLCQAHGLTPEQVVAIASHDGGKQALETVQRLLPVLCQAHGLTPEQVVAIASHDGGKQALETVQRLLPVLCQAHGLTPEQVVAIASNNGGKQALETVQRLLPVLCQAHGLTPEQVVAIASNGGGKQALETVQRLLPVLCQAHGLTPEQVVAIASNNGGKQALETVQRLLPVLCQAHGLTPEQVVAIASHDGGKQALETVQRLLPVLCQAHGLTPEQVVAIASNIGGKQALETVQRLLPVLCQAHGLTPEQVVAIASHDGGKQALE**SIVAQLSRPDPSLAALTGSSEVEFSHEYWMRHALTLAKRARDEREVPVGAVLVLNNRVIGEGWNRAIGLHDPTAHAEIMALRQGGLVMQNYRLIDATLYVTFEPCVMCAGAMIHSRIGRVVFGVRNSKRGAAGSLMNVLNYPGMNHRVEITEGILADECAALLCDFYRMPRQVFNAQKKAQSSINSGGSYALGPYQISAPQLPAYNGQTVGTFYYVNDAGGLESKVFSSGGPTPYPNYANAGHVEGQSALFMRDNGISEGLVFHNNPEGTCGFCVNMTETLLPENAKMTVVPPEG*

**pTABE_v6** (from N- to C-terminus):

bpNLS-N-terminal domain-**TALE repeats**-C-terminal domain-GS linker-TadA8e-SGGS linker-DddA6 C half

bpNLS-N-terminal domain-**TALE repeats**-C-terminal domain-GS linker-TadA8e-SG linker-DddA6 N half

MKRTADGSEFESPKKKRKVMAHHTEAATGEWDEVQSGLRAADAPPPTMRVAVTAARPPRAKPAPRRRAAQPSDASPAAQVDLRTLGYSQQQQEKIKPKVRSTVAQHHEALVGHGFTHAHIVALSQHPAALGTVAVKYQDMIAALPEATHEAIVGVGKQWSGARALEALLTVAGELRGPPLQLDTGQLLKIAKRGGVTAVEAVHAWRNALTGAPLN**LTPEQVVAIASNNGGKQALETVQRLLPVLCQAHGLTPEQVVAIASHDGGKQALETVQRLLPVLCQAHGLTPEQVVAIASHDGGKQALETVQRLLPVLCQAHGLTPEQVVAIASNIGGKQALETVQRLLPVLCQAHGLTPEQVVAIASNIGGKQALETVQRLLPVLCQAHGLTPEQVVAIASHDGGKQALETVQRLLPVLCQAHGLTPEQVVAIASNNGGKQALETVQRLLPVLCQAHGLTPEQVVAIASNIGGKQALETVQRLLPVLCQAHGLTPEQVVAIASNIGGKQALETVQRLLPVLCQAHGLTPEQVVAIASHDGGKQALETVQRLLPVLCQAHGLTPEQVVAIASHDGGKQALETVQRLLPVLCQAHGLTPEQVVAIASNNGGKQALETVQRLLPVLCQAHGLTPEQVVAIASNNGGKQALETVQRLLPVLCQAHGLTPEQVVAIASNIGGKQALETVQRLLPVLCQAHGLTPEQVVAIASNGGGKQALETVQRLLPVLCQAHGLTPEQVVAIASNIGGKQALE**SIVAQLSRPDPSLAALTGSSEVEFSHEYWMRHALTLAKRARDEREVPVGAVLVLNNRVIGEGWNRAIGLHDPTAHAEIMALRQGGLVMQNYRLIDATLYVTFEPCVMCAGAMIHSRIGRVVFGVRNSKRGAAGSLMNVLNYPGMNHRVEITEGILADECAALLCDFYRMPRQVFNAQKKAQSSINSGGSAIPVKRGATGETKVFIGNSNSPKSPTKGGC*

MKRTADGSEFESPKKKRKVMAHHTEAATGEWDEVQSGLRAADAPPPTMRVAVTAARPPRAKPAPRRRAAQPSDASPAAQVDLRTLGYSQQQQEKIKPKVRSTVAQHHEALVGHGFTHAHIVALSQHPAALGTVAVKYQDMIAALPEATHEAIVGVGKQWSGARALEALLTVAGELRGPPLQLDTGQLLKIAKRGGVTAVEAVHAWRNALTGAPLN**LTPEQVVAIASNIGGKQALETVQRLLPVLCQAHGLTPEQVVAIASNGGGKQALETVQRLLPVLCQAHGLTPEQVVAIASNGGGKQALETVQRLLPVLCQAHGLTPEQVVAIASHDGGKQALETVQRLLPVLCQAHGLTPEQVVAIASHDGGKQALETVQRLLPVLCQAHGLTPEQVVAIASHDGGKQALETVQRLLPVLCQAHGLTPEQVVAIASNNGGKQALETVQRLLPVLCQAHGLTPEQVVAIASNGGGKQALETVQRLLPVLCQAHGLTPEQVVAIASNNGGKQALETVQRLLPVLCQAHGLTPEQVVAIASHDGGKQALETVQRLLPVLCQAHGLTPEQVVAIASNIGGKQALETVQRLLPVLCQAHGLTPEQVVAIASHDGGKQALE**SIVAQLSRPDPSLAALTGSSEVEFSHEYWMRHALTLAKRARDEREVPVGAVLVLNNRVIGEGWNRAIGLHDPTAHAEIMALRQGGLVMQNYRLIDATLYVTFEPCVMCAGAMIHSRIGRVVFGVRNSKRGAAGSLMNVLNYPGMNHRVEITEGILADECAALLCDFYRMPRQVFNAQKKAQSSINSGGSYALGPYQISAPQLPAYNGRTVGTFYYVNDAGGLESKVFISGGPTPYPNYANAGHVEGQSALFMRDNGISEGLVFHNNPEGTCGFCVNMIETLLPENAKMTVVPPEG*

**DdCBE** (from N- to C-terminus):

bpNLS-N-terminal domain-**TALE repeats**-C-terminal domain-GS linker-DddA C half-SGGS linker-UGI-SGGS linker-NLS

bpNLS-N-terminal domain-**TALE repeats**-C-terminal domain-GS linker-DddA N half-SGGS linker-UGI-SGGS linker-NLS

MKRTADGSEFESPKKKRKVMAHHTEAATGEWDEVQSGLRAADAPPPTMRVAVTAARPPRAKPAPRRRAAQPSDASPAAQVDLRTLGYSQQQQEKIKPKVRSTVAQHHEALVGHGFTHAHIVALSQHPAALGTVAVKYQDMIAALPEATHEAIVGVGKQWSGARALEALLTVAGELRGPPLQLDTGQLLKIAKRGGVTAVEAVHAWRNALTGAPLN**LTPEQVVAIASNIGGKQALETVQRLLPVLCQAHGLTPEQVVAIASNIGGKQALETVQRLLPVLCQAHGLTPEQVVAIASHDGGKQALETVQRLLPVLCQAHGLTPEQVVAIASNNGGKQALETVQRLLPVLCQAHGLTPEQVVAIASNGGGKQALETVQRLLPVLCQAHGLTPEQVVAIASNIGGKQALETVQRLLPVLCQAHGLTPEQVVAIASNGGGKQALETVQRLLPVLCQAHGLTPEQVVAIASHDGGKQALETVQRLLPVLCQAHGLTPEQVVAIASHDGGKQALETVQRLLPVLCQAHGLTPEQVVAIASNIGGKQALETVQRLLPVLCQAHGLTPEQVVAIASHDGGKQALETVQRLLPVLCQAHGLTPEQVVAIASNNGGKQALETVQRLLPVLCQAHGLTPEQVVAIASHDGGKQALETVQRLLPVLCQAHGLTPEQVVAIASHDGGKQALE**SIVAQLSRPDPSLAALTGSAIPVKRGATGETKVFTGNSNSPKSPTKGGCSGGSTNLSDIIEKETGKQLVIQESILMLPEEVEEVIGNKPESDILVHTAYDESTDENVMLLTSDAPEYKPWALVIQDSNGENKIKMLSGGSPKKKRKV*

MKRTADGSEFESPKKKRKVMAHHTEAATGEWDEVQSGLRAADAPPPTMRVAVTAARPPRAKPAPRRRAAQPSDASPAAQVDLRTLGYSQQQQEKIKPKVRSTVAQHHEALVGHGFTHAHIVALSQHPAALGTVAVKYQDMIAALPEATHEAIVGVGKQWSGARALEALLTVAGELRGPPLQLDTGQLLKIAKRGGVTAVEAVHAWRNALTGAPLN**LTPEQVVAIASHDGGKQALETVQRLLPVLCQAHGLTPEQVVAIASNIGGKQALETVQRLLPVLCQAHGLTPEQVVAIASNNGGKQALETVQRLLPVLCQAHGLTPEQVVAIASHDGGKQALETVQRLLPVLCQAHGLTPEQVVAIASHDGGKQALETVQRLLPVLCQAHGLTPEQVVAIASNNGGKQALETVQRLLPVLCQAHGLTPEQVVAIASNIGGKQALETVQRLLPVLCQAHGLTPEQVVAIASNGGGKQALETVQRLLPVLCQAHGLTPEQVVAIASNGGGKQALETVQRLLPVLCQAHGLTPEQVVAIASNIGGKQALETVQRLLPVLCQAHGLTPEQVVAIASNGGGKQALETVQRLLPVLCQAHGLTPEQVVAIASHDGGKQALETVQRLLPVLCQAHGLTPEQVVAIASNIGGKQALETVQRLLPVLCQAHGLTPEQVVAIASNGGGKQALETVQRLLPVLCQAHGLTPEQVVAIASHDGGKQALE**SIVAQLSRPDPSLAALTGSGSYALGPYQISAPQLPAYNGQTVGTFYYVNDAGGLESKVFSSGGPTPYPNYANAGHVEGQSALFMRDNGISEGLVFHNNPEGTCGFCVNMTETLLPENAKMTVVPPEGSGGSTNLSDIIEKETGKQLVIQESILMLPEEVEEVIGNKPESDILVHTAYDESTDENVMLLTSDAPEYKPWALVIQDSNGENKIKMLSGGSPKKKRKV*

**DddA^E1347A^ / A3A^Y130F^** (from N- to C-terminus):

bpNLS-N-terminal domain-**TALE repeats**-C-terminal domain-GSGGS linker-NLS-A3A^Y130F^-SGGS linker-UGI-SGGS linker-NLS

bpNLS-N-terminal domain-**TALE repeats**-C-terminal domain-GSSGGS linker-DddA^E1347A^

MKRTADGSEFESPKKKRKVMAHHTEAATGEWDEVQSGLRAADAPPPTMRVAVTAARPPRAKPAPRRRAAQPSDASPAAQVDLRTLGYSQQQQEKIKPKVRSTVAQHHEALVGHGFTHAHIVALSQHPAALGTVAVKYQDMIAALPEATHEAIVGVGKQWSGARALEALLTVAGELRGPPLQLDTGQLLKIAKRGGVTAVEAVHAWRNALTGAPLN**LTPEQVVAIASNIGGKQALETVQRLLPVLCQAHGLTPEQVVAIASNIGGKQALETVQRLLPVLCQAHGLTPEQVVAIASHDGGKQALETVQRLLPVLCQAHGLTPEQVVAIASNNGGKQALETVQRLLPVLCQAHGLTPEQVVAIASNGGGKQALETVQRLLPVLCQAHGLTPEQVVAIASNIGGKQALETVQRLLPVLCQAHGLTPEQVVAIASNGGGKQALETVQRLLPVLCQAHGLTPEQVVAIASHDGGKQALETVQRLLPVLCQAHGLTPEQVVAIASHDGGKQALETVQRLLPVLCQAHGLTPEQVVAIASNIGGKQALETVQRLLPVLCQAHGLTPEQVVAIASHDGGKQALETVQRLLPVLCQAHGLTPEQVVAIASNNGGKQALE**SIVAQLSRPDPSLAALTGSGGSEASPASGPRHLMDPHIFTSNFNNGIGRHKTYLCYEVERLDNGTSVKMDQHRGFLHNQAKNLLCGFYGRHAELRFLDLVPSLQLDPAQIYRVTWFISWSPCFSWGCAGEVRAFLQENTHVRLRIFAARIFDYDPLYKEALQMLRDAGAQVSIMTYDEFKHCWDTFVDHQGCPFQPWDGLDEHSQALSGRLRAILQNQGNSGGSTNLSDIIEKETGKQLVIQESILMLPEEVEEVIGNKPESDILVHTAYDESTDENVMLLTSDAPEYKPWALVIQDSNGENKIKMLSGGSPKKKRKV*

MKRTADGSEFESPKKKRKVMAHHTEAATGEWDEVQSGLRAADAPPPTMRVAVTAARPPRAKPAPRRRAAQPSDASPAAQVDLRTLGYSQQQQEKIKPKVRSTVAQHHEALVGHGFTHAHIVALSQHPAALGTVAVKYQDMIAALPEATHEAIVGVGKQWSGARALEALLTVAGELRGPPLQLDTGQLLKIAKRGGVTAVEAVHAWRNALTGAPLN**LTPEQVVAIASHDGGKQALETVQRLLPVLCQAHGLTPEQVVAIASNIGGKQALETVQRLLPVLCQAHGLTPEQVVAIASNNGGKQALETVQRLLPVLCQAHGLTPEQVVAIASHDGGKQALETVQRLLPVLCQAHGLTPEQVVAIASHDGGKQALETVQRLLPVLCQAHGLTPEQVVAIASNNGGKQALETVQRLLPVLCQAHGLTPEQVVAIASNIGGKQALETVQRLLPVLCQAHGLTPEQVVAIASNGGGKQALETVQRLLPVLCQAHGLTPEQVVAIASNGGGKQALETVQRLLPVLCQAHGLTPEQVVAIASNIGGKQALETVQRLLPVLCQAHGLTPEQVVAIASNGGGKQALETVQRLLPVLCQAHGLTPEQVVAIASHDGGKQALETVQRLLPVLCQAHGLTPEQVVAIASNIGGKQALETVQRLLPVLCQAHGLTPEQVVAIASNGGGKQALETVQRLLPVLCQAHGLTPEQVVAIASHDGGKQALE**SIVAQLSRPDPSLAALTGSSGGSGSYALGPYQISAPQLPAYNGQTVGTFYYVNDAGGLESKVFSSGGPTPYPNYANAGHVAGQSALFMRDNGISEGLVFHNNPEGTCGFCVNMTETLLPENAKMTVVPPEGAIPVKRGATGETKVFTGNSNSPKSPTKGGC*
